# Design, Synthesis, Molecular Docking, and Biological Evaluation of Pyrazole Hybrid Chalcone Conjugates as Potential Anticancer Agents and Tubulin Polymerization Inhibitors

**DOI:** 10.3390/ph15030280

**Published:** 2022-02-24

**Authors:** Md. Jahangir Alam, Ozair Alam, Ahmad Perwez, Moshahid Alam Rizvi, Mohd Javed Naim, Vegi G. M. Naidu, Mohd Imran, Mohammed M. Ghoneim, Sultan Alshehri, Faiyaz Shakeel

**Affiliations:** 1 Medicinal Chemistry and Molecular Modeling Lab, Department of Pharmaceutical Chemistry, School of Pharmaceutical Education and Research, Jamia Hamdard, New Delhi 110062, India; zahangir.jh@gmail.com (M.J.A.); javednaim88@rediffmail.com (M.J.N.); 2 Genome Biology Lab, Department of Biosciences, Jamia Millia Islamia, New Delhi 110020, India; ahmad86.delhi@gmail.com (A.P.); rizvi_ma@yahoo.com (M.A.R.); 3 National Institute of Pharmaceutical Education and Research, Guwahati 781101, India; vgmnaidu@gmail.com; 4 Department of Pharmaceutical Chemistry, Faculty of Pharmacy, Northern Border University, Rafha 91911, Saudi Arabia; imran.pchem@gmail.com; 5 Department of Pharmacy Practice, College of Pharmacy, Al-Maarefa University, Ad Diriyah 13713, Saudi Arabia; mghoneim@mcst.edu.sa; 6 Department of Pharmaceutics, College of Pharmacy, King Saud University, Riyadh 11451, Saudi Arabia; salshehri1@ksu.edu.sa (S.A.); fsahmad@ksu.esu.sa (F.S.)

**Keywords:** pyrazole conjugate, anticancer, MTT assay, molecular docking, cytotoxicity, tubulin polymerization inhibitors

## Abstract

Some (*E*)-3-(3-(4-(benzyloxy)phenyl)-1-phenyl-1*H*-pyrazol-4-yl)-1-phenylprop-2-en-1-one conjugates **5a**–**r** were designed; synthesized; characterized by ^1^H, ^13^C NMR, and ESI-MS; and evaluated for tubulin polymerization inhibitory activity and in vitro cytotoxicity against breast (MCF-7), cervical (SiHa), and prostate (PC-3) cancer cell lines, as well as a normal cell line (HEK-293T). The compounds were also tested to determine their binding modes at the colchicine-binding site of tubulin protein (PDB ID-3E22), for in silico ADME prediction, for bioactivity study, and for PASS prediction studies. Among all the synthesized conjugates, compound **5o** exhibited excellent cytotoxicity with an IC_50_ value of 2.13 ± 0.80 µM (MCF-7), 4.34 ± 0.98 µM (SiHa), and 4.46 ± 0.53 µM (PC-3) against cancer cell lines. The compound did not exhibit significant toxicity to the HEK cells. Results of the in silico prediction revealed that the majority of the conjugates possessed drug-like properties.

## 1. Introduction

Cancer is significantly the most common health issue worldwide. It is considered the second most recurrent death-causing factor after cardiovascular disorders [1,2]. It accounts for nearly 10 million deaths in 2020, with most deaths due to lung cancer, followed by colon, liver, stomach, and breast cancer [3]. The World Health Organization (WHO) has projected that by 2030, more than 13 million annual deaths are expected to occur from cancer globally [4]. Currently available chemotherapeutic agents that target cell division and angiogenesis, or induce cancer cell death through different signaling pathways comprise the major treatment options for cancer. While these therapies are effective in treating early-stage cancers, the efficacy against advanced cancers, especially multidrug-resistant cancers, is limited. Furthermore, these classes of drug molecules face numerous restrictions such as greater systemic toxicity and complex synthesis [5].

**Figure 1:**
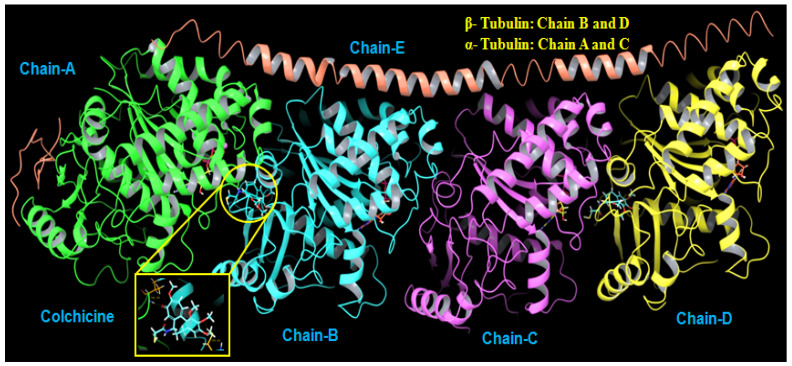
Colchicine-binding site (PDB: 1SA0).

The microtubules are a dynamic polymeric network of two closely related 55 kDa proteins in cells known as α and β-tubulin dimers [6]. It is responsible for the formation of the mitotic spindle during cell division and the severance of duplicated chromosomes. It also plays a crucial role as an important target for cancer therapy because of its essential role in cell proliferation and angiogenesis [7]. Since rapidly dividing cancer cells are highly dependent on tubulin polymerization/depolymerization, interfering with tubulin dynamics has become an important approach for the development of mitotic inhibitors [8,9]. The tubulin inhibitors have been classified based on their binding domains to tubulin *viz*. microtubule destabilizers (e.g., Combretastatin A-4) [10], colchicine site-binding agents (e.g., colchicine and podophyllotoxin) [11,12], and the vinca site-binding agent (e.g., vinblastine and vincristine) [13]. The microtubule disrupters inhibit tubulin polymerization and consequently interfere with the formation of the necessary mitotic assembly required for cell division. On the other hand, microtubule stabilizers, such as taxanes [14], paclitaxel, docetaxel, and laulimalide, prevent the depolymerization of microtubules [15]. Both agents, which can disrupt as well as stabilize microtubules, have found clinical success as anticancer agents, therefore the compounds that can affect tubulin or microtubule stability may show potential against various cancers. That is why, nowadays, scientists draw attention to the drug discovery of microtubule-targeting agents/inhibitors for anticancer therapy. The trimethoxy/dimethoxy phenyl rings of reported ligands are well-defined pharmacophores that bind to the interface of the α-tubulin and β-tubulin of the colchicine-binding site, which destabilizes the microtubules [16,17,18,19]. In the past few years, several chalcone derivatives were found as the most effective tubulin polymerization inhibitors as well as safer anticancer molecules [20]. The three binding sites are present in tubulin as the colchicine-binding site, vinca-binding site, and taxane-binding site. The tubulin polymerization inhibitors are docked into the colchicine-binding site of the tubulin protein. The colchicines always interact and bind with the α-tubulin and β-tubulin interface, which was also established by molecular docking [21] (Figure 1). The colchicine forms hydrogen, bonding with the Cys-241 of the β subunit of tubulin.

**Figure 2:**
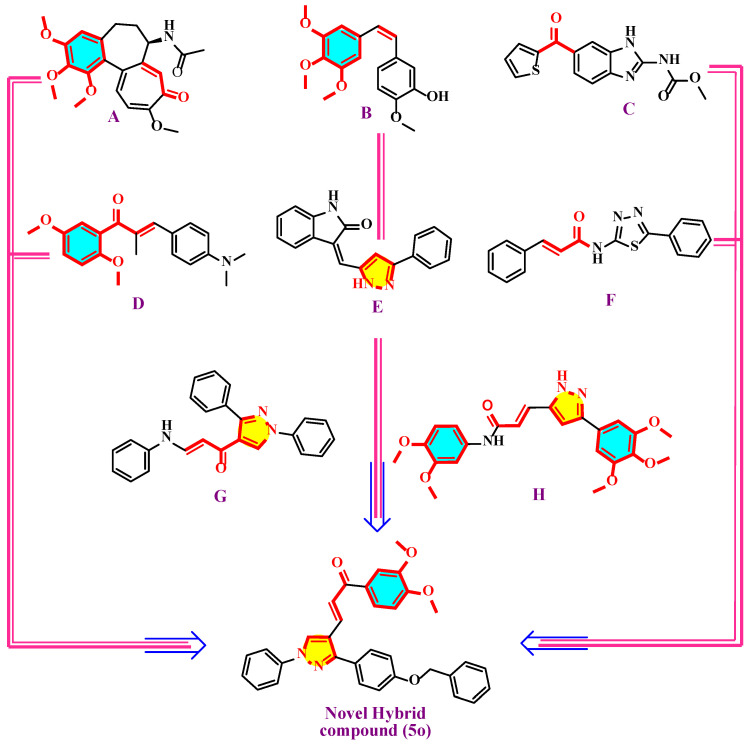
Structures of some potential cytotoxic agents and tubulin polymerization inhibitors: colchicine (**A**), combretastatin-A4 (**B**), nocodazole (**C**), MDL-27048 (**D**), reported pyrazole-linked conjugates (**E**), reported chalcone-linked conjugates (**F**), reported pyrazole-linked chalcone conjugates (**G**,**H**), and novel hybrid compound (**5o**).

Pyrazole is a significant class of heterocyclic compounds in pharmaceutical chemistry. Many pyrazole-based lead compounds have been evaluated for their biological effects, including anti-inflammatory, antimicrobial, antioxidant, anti-depressant, and anti-influenza activities [22,23]. Several recent reports suggested that pyrazole derivatives have shown promising anticancer activity, indicating their use in the development of new anticancer agents [24]. Among the anticancer pyrazole moieties, 1,3-diphenyl pyrazole derivatives have been shown as potent and effective cytotoxic agents [25]. Chalcone conjugates have shown wide pharmacological efficacy as well as synthetic applications in pharmaceutical chemistry [26,27]. Presently, colchicine (A), combretastatin A-4 (B) analogs, pyrazole linker chalcone congeners, and the marketed drug nocodazole (C) are the most important tubulin-binding agents that inhibit tubulin depolymerization [28]. In the recently reported literature, Peyrot et al. synthesized (*E*)-3-(4-(dimethylamino)phenyl)-1-(2,5-dimethoxyphenyl)-2-methylprop-2-en-1-one (D, MDL-27048) [29] as anti-mitotic agents with rapid and reversible binding to the colchicine-binding site for inhibiting its assembly to microtubules. Kamal et al. [30] designed and synthesized a novel scaffold (*Z*)-3-((3-phenyl-1*H*-pyrazol-5-yl)methylene)indolin-2-one (E), which has significant polymerization inhibitory activity. Moreover, various 1,3,4 thiadiazole ring-based cinnamide derivatives’ tubulin (F) [31] also exhibited significant growth inhibition effects against MCF-7 and A549 cell lines apart from effectively inhibiting tubulin polymerization. Srinivasa and co-workers synthesized a new class of (*Z*)-1-(1,3-diphenyl-1*H*-pyrazol-4-yl)-3-(phenylamino)prop-2-en-1-one derivatives (G) as an effective anticancer agent with IC_50_ values ranging from 1.25 to 3.98 µM [32]. In addition, Kamal and co-workers synthesized a series of pyrazole-linked arylcinnamides (H) as potential antiproliferative agents with significant tubulin polymerization inhibitory action (Figure 2) [33].

Hybrid chalcone derivatives possess structural similarities to occurring moieties, such as Combretastatin A-4, obtained from the bark of *Combretum caffrum* [34]. It has exhibited cytotoxicity against a broad range of human cancer cell lines by binding at the colchicine-binding site on β tubulin with a percentage of inhibition in the range of 28.42–66.40%. Furthermore, hybrid chalcones linked to other heterocyclic moieties have resulted in enhanced anticancer activity [35]. With this background, the present work was carried out to design and synthesize novel pyrazole-linked hybrid chalcone conjugates, and to evaluate their ability to inhibit tubulin polymerization and consequently prevent cancer growth.

## 2. Results and Discussion

### 2.1. Chemistry

**Scheme 1:**
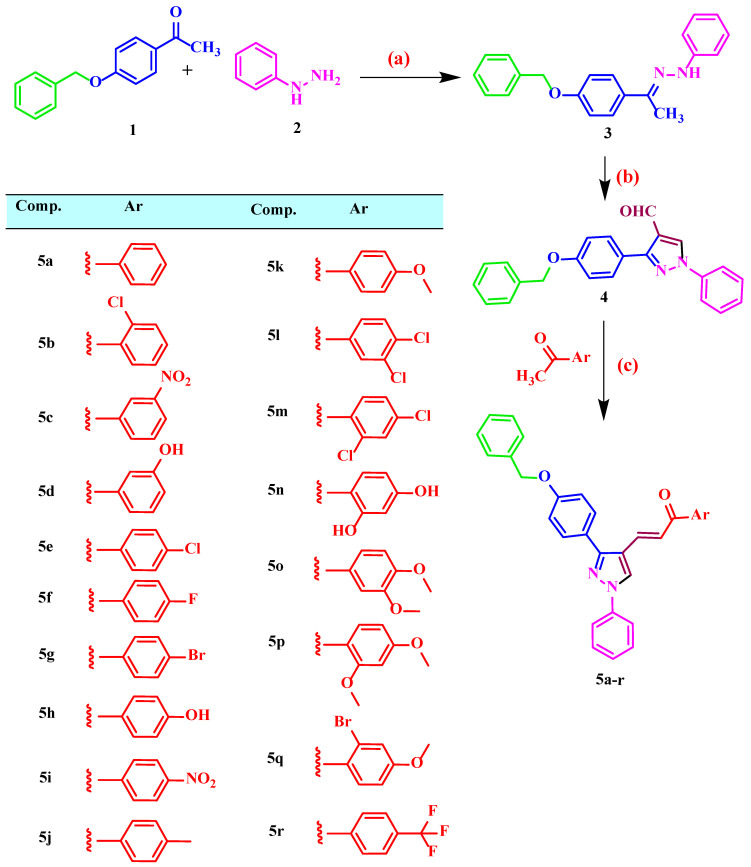
General synthesis of (*E*)-3-(3-(4-(benzyloxy)phenyl)-1-phenyl-1*H*-pyrazol-4-yl)-1-phenylprop-2-en-1-one derivatives (**5a**–**r**). Reagents and conditions: (**a**) absolute ethanol and glacial acetic acid, 50–60 °C, refluxed for 6–8 h; (**b**) DMF and POCl_3_, heated for 2 h on steam bath; and (**c**) absolute ethanol, 40% NaOH, reflux.

The protocol for the synthesis of analog (**5a**–**r**) is described in Scheme 1. Compound **1** was prepared by reacting 4-hydroxyacetophenone and benzyl chloride. It was then reacted with phenylhydrazine (**2**) to give compound **3**, which was further cyclized in the presence of POCl_3_ and DMF (Vilsmeier Haack reaction) to give 3-(4-(Benzyloxy) phenyl)-1-phenyl-1*H*-pyrazole-4-carbaldehyde (**4**). Equimolar quantities of compound **4** and various substituted acetophenones underwent Claisen–Schmidt condensation to yield the final pyrazole–chalcone conjugates. All compounds were characterized by FT-IR, ^1^H NMR, ^13^C NMR, and mass spectral data. The ^1^H NMR, ^13^C NMR, and mass spectra of lead compounds are presented in Figures S1–S14. The ^1^H NMR spectra of the aldehydic proton in compound **4** appeared as a singlet at δ 9.82 ppm. The H-6 and H-7 of compound **5a** appeared as a doublet at δ 7.95 and δ 8.01 ppm, respectively, with the coupling constant (*J* = 15.6–15.7 Hz), which agrees with the trans-configuration of α,β-unsaturated ketone and the disappearance of the signal corresponding to the C-H of aldehyde. Moreover, in the ^13^C NMR spectrum, the signal for C-1 was in the range of δ 127.8–139.6 ppm and the signal for C-7 appeared at δ 113.1–127.7 ppm, while the C=O carbon atom appeared at δ 186.3–196.1 ppm. The IR spectra showed absorption bands at 1755–1675, 1653–1590, and 1590–1512 cm^−1^, corresponding to the C=O, C=N, and C=C functionalities, respectively. Mass spectra showed a molecular ion peak, i.e., (*m*/*z*) [M^+^] with a characteristic fragmentation pattern involving the loss of the phenyl group in all cases.

### 2.2. Molecular Docking Study

**Figure 3:**
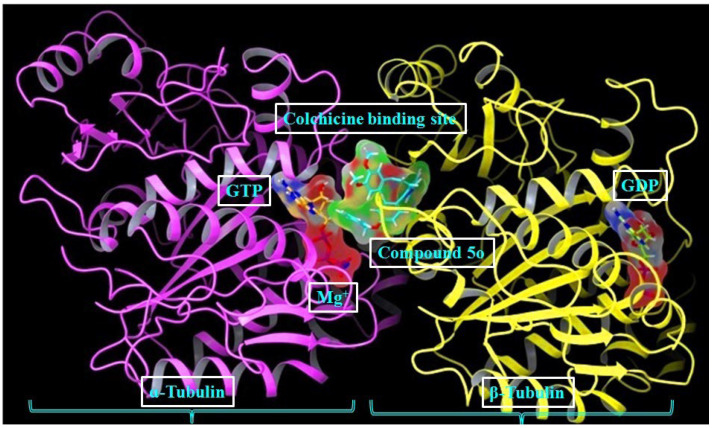
Different binding sites of tubulin protein complexes with **5o** (turquoise-color stick model), GTP, Mg^+2^, and GDP. The α-chain is represented in purple color and the β-chain is represented in yellow color.

**Figure 4:**
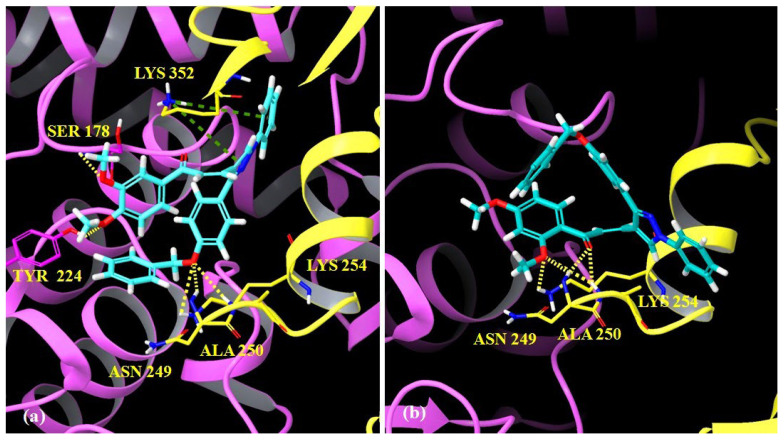
(**a**) Docked pose of compound **5o** (turquoise color) showing hydrogen bond interaction (yellow dashed lines) with ASN 249, ALA 250, LYS 254, SER 178, and TYR 224; pi-cation interaction with LYS 352 in the colchicine-binding site of tubulin. (**b**) Docked pose of compound **5p** (turquoise color) showing hydrogen bond interaction (yellow dashed lines) with ASN 249, ALA 250, and LYS 254 in the colchicine-binding site of tubulin. The α-chain is represented in a purple color cartoon and the β-chain is represented in a yellow color cartoon model.

**Figure 5:**
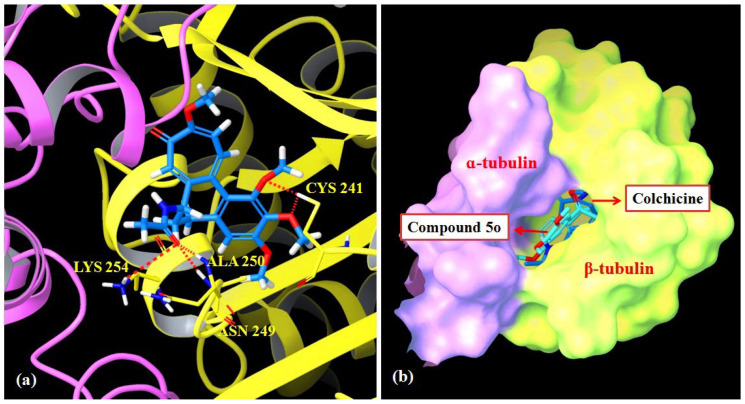
(**a**) Docked pose of colchicine (blue color) showing hydrogen bond interaction (red dashed lines) with ASN 249, ALA 250, LYS 254, and CYS 241 in the colchicine-binding site of tubulin. The α-chain is represented in a purple color cartoon and the β-chain is represented in a yellow color cartoon model. (**b**) Receptor surface of superimposing docked pose of compound **5o** (turquoise-color stick model) with colchicine (blue-color stick model) at colchicine-binding site of tubulin. The α-chain is represented in the purple color surface and the β-chain is represented in the yellow color surface.

Molecular docking was performed to establish the binding ability of the newer synthesized compounds to the colchicine-binding site of tubulin (PDB code: 3E22). The docking scores of the compounds (**5a**–**r**) and co-crystal ligand colchicine are given in Table 1. The protein backbone of tubulin is depicted in purple and yellow colors for α and β chains, respectively. The favorable hydrogen bonding interactions are shown with yellow dashed lines and the amino acid residues that interact with the compounds are represented as purple and yellow tubes. The standard compounds were docked as co-crystal ligands at the colchicine-binding site of tubulin. Docking studies revealed that eighteen newer compounds (5a–r) exhibited a higher docking score than that of the co-crystal ligand, suggesting that the synthesized molecules have better interaction and better accommodation within the binding site. The different binding sites of tubulin protein complexes with **5o** viz. GTP, Mg^+2^, and GDP are shown in Figure 3. The 3D docked images of compound **5o** and **5p** complexes with tubulin are represented in Figure 4. The most promising compound **5o** could fit well into the colchicine-binding site of the tubulin, making five hydrogen bond interactions with the most important active site residues ASN 249 (O....H_2_N, 3.53 Å, β-), ALA 250 (O....H_2_N, 3.70 Å) and LYS 254 (O....H_3_N^+^, 3.30 Å), SER 178 (O....HN, 3.65 Å), and TYR 224 (O....HO, 3.23 Å) pi-cation LYS 352 (pyrazole....H_3_N^+^, 6.17 Å, and phenyl....H_3_N^+^, 6.26 Å), as depicted in Figure 5a. The methoxy group of the 3,4-dimethoxy aryl moiety exhibited interaction with the side chain of SER 178 (O....H_2_N, 1.93 Å) and TYR 224 (O....H_2_N, 1.93 Å) in a similar manner as the co-crystal ligand colchicine. Introduction of a methoxy group at positions two and four of the aromatic ring of compounds leads to a slight decrease in the inhibitory activity primarily due to the changing of the orientation of the 2,4-dimethoxy group in the binding site, as observed for compound **5p**. The carbonyl group of **5p** also showed hydrogen bond interaction with the side chain of ASN 249 (O....H_2_N, 2.87 Å), ALA 250 (O....H_2_N, 2.91 Å), and LYS 254 (O....H_3_N^+^, 3.10 Å).

The present study showed that the presence of methoxy groups at positions three and four of the aromatic ring of the ligand has a significant inhibitory effect on the tubulin polymerization at positions two and four. The positions of the methoxy (OCH_3_) groups in these two (**5o** and **5p**) compounds showed slight differences in the in silico as well as in vitro studies. This could be due to the difference in the position of the substituents present in the aryl rings. These observations also provide a possible explanation as to why the 3,4-dimethoxy derivative **5o** is the only compound from the series which shows high affinity as well as a high docking score. The docked compound **5o** was superimposed with co-crystal ligand colchicine and its binding pattern was then compared (Figure 5a). The co-crystal ligand colchicine formed four hydrogen bond interactions with the most important active site residues ASN 249 (O....H_2_N, 1.76 Å), ALA 250 (O....H_2_N, 1.95 Å), LYS 254 (O....H_3_N^+^, 3.61 Å), and CYS 241 (O....HS, 2.40 Å and 2.61 Å). The receptor surface of the docked conformer **5o** complex with tubulin is presented in Figure 5b. The dG binding energy of the compounds was found in the range of −48.34 to −91.43 Kcal/mol for the receptor sites (Table 1). All compounds displayed a higher binding free energy (dG bond) for tubulin, which indicates that the compounds may have higher affinity and stability for the tubulin protein. To validate the molecular docking results, the synthesized compounds with higher dock scores were screened by the in vitro tubulin inhibitory assay. The outcome of molecular docking studies, along with the biological activity, revealed that compound **5o** was the most potent inhibitor.

### 2.3. Pharmacological Activities

#### 2.3.1. In Vitro Cytotoxicity Activity

**Figure 6:**
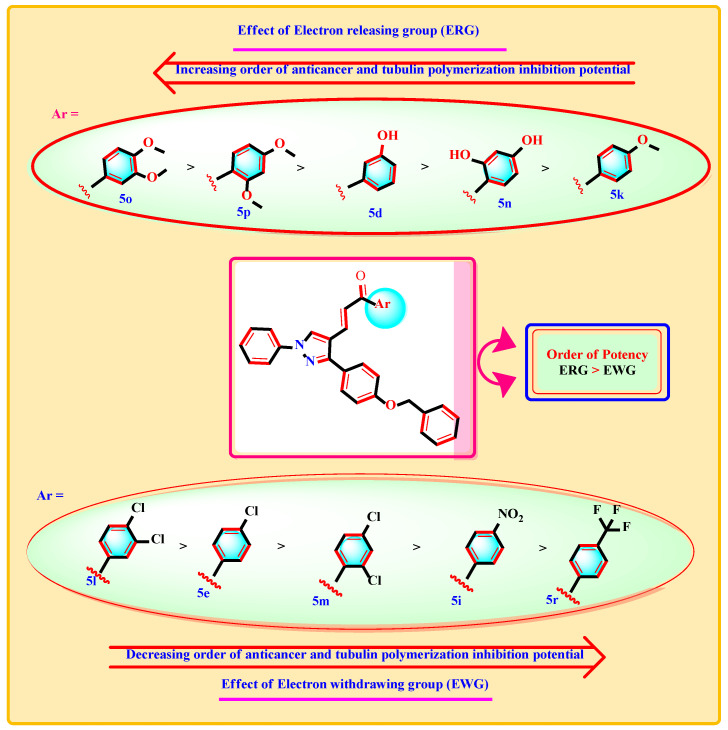
SAR of titled compounds.

The resultant compounds were tested for their in vitro cytotoxic activity by MTT assay against breast (MCF-7), cervical (SiHa), and prostate (PC-3) cancer cell lines, and the human embryonic kidney cell line (HEK) [36]. The relative absorbance of the treated versus control (untreated) cells was used in determining the percentage viability and the IC_50_ value of Combretastatin A4 (CA4), which was used as the standard. IC_50_ values of various compounds in the MTT assay are presented in Table 2. Almost all synthesized conjugates were selectively cytotoxic against breast MCF-7 cancerous cells. Moreover, compounds **5d**, **5o**, and **5p** displayed pronounced growth inhibition on MCF-7 cells with IC_50_ values in the range of 2.13–4.10 µM, which were equivalent or better than the standard CA-4 (4.12–5.23 µM). The effective growth inhibitory activity was exhibited by compound **5o** (IC_50_ −2.13 ± 0.80 µM), followed by the compounds **5p** (IC_50_ 3.45 ± 1.28 µM) and **5d** (IC_50_ 4.10 ± 1.12 µM) against MCF-7 cancer cells. The other two cancer cell lines, SiHa and PC-3, also showed maximum sensitivity towards these two compounds with IC_50_ values of less than 6.52 µM. Moreover, compound **5n** showed higher potency against SiHa cells with an IC_50_ value of 3.60 ± 0.45 µM, while compound **5d** revealed selective potency against PC-3 cells with an IC_50_ value of 2.97 ± 0.88 µM. All compounds were further tested on non-cancerous HEK cells, where most compounds did not show any significant cytotoxicity (IC_50_ > 50 µM). The compounds **5r** and **5i** displayed moderate growth inhibition on HEK cells with IC_50_ values of 38.30 and 45.23 µM, respectively. These results expressed the selectivity of 1,3-diphenyl-1*H* pyrazole hybrids towards cancer cells compared to normal HEK cells. Therefore, the high cytotoxicity of the synthesized anticancer agents and their selectivity towards cancer cells were found to be characteristic factors. Subsequently, SAR studies were done by investigating the effect of substituents on cytotoxic activity. It has been shown that compounds with strong electron-donating groups on the aromatic ring (OH > OCH_3_) exhibited potent cytotoxic activity. Furthermore, the compounds with electron-donating groups such as 3,4-dimethoxy substitution and 2,4-dimethoxy (**5o** and **5p**) exhibited higher potency than *para*-Methoxy substitutions (**5k**). Furthermore, compounds with electron-withdrawing groups such as para-chloro, para-nitro, 3,4-dichloro, and trifluoromethyl substitutions (**5e**, **5i**, **5l**, and **5r**) on the phenyl rings displayed good cytotoxic strength (Cl > NO_2_ > CF_3_) on cancerous cells (Figure 6). The majority of the compounds in this scheme exhibited moderate selectivity index values. Moreover, the pharmacological efficacy of the synthesized entities **5d**, **5n**, **5o**, and **5p** has created a scope to explore their profound efficacy at the cellular level and particularly using the mechanism proposed for cytotoxic effects.

#### 2.3.2. Tubulin Polymerization Assay

**Figure 7:**
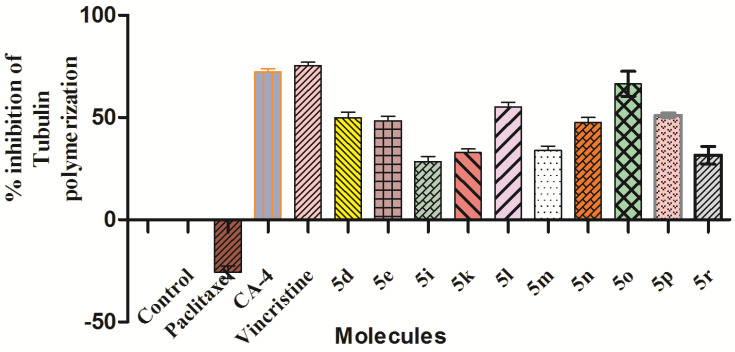
Effect of conjugates on in vitro tubulin polymerization. Values indicated are the mean ± SD of two different experiments performed in triplicates.

The α and β tubulin subunits are recognized by heterodimers and self-assemble into a stable microtubule in a time-dependent manner. Assessment of the inhibitory effect of new pyrazolic chalcone conjugates on tubulin polymerization was also carried out. The progression of tubulin polymerization was thus examined by monitoring the increase in the fluorescence intensity at 450 nm (excitation wavelength of 360 nm) in a 384-plate for 1 h at 37 °C with and without the conjugate at 10 µM concentration. CA-4 (6 µM) and Vincristine (3 µM) were used as the positive control, whereas paclitaxel (3 µM) was used as the negative control. The effect of lead conjugates on tubulin polymerization is presented in Figure 5. The compounds **5d**, **5e**, **5i**, **5k**, **5l**, **5m**, **5n**, **5o**, **5p**, and **5r** exhibited 28.42–66.40% inhibition on tubulin polymerization (Figure 7). Among these compounds, **5o**, **5l**, and **5p** showed significant inhibition of tubulin polymerization with IC_50_ values of 1.15, 1.65, and 1.95 μM, respectively, as compared with 1.46 μM for Combretastatin A-4, as shown in Table 1. In general, these outcomes imply that the lead molecules **5o**, **5l**, and **5p** possess a potent inhibitory effect on microtubule assembly in vitro.

### 2.4. In Silico Computational Studies

Physicochemical properties of lead compounds are important for their development as anticancer agents with an inhibitory effect on tubulin polymerization agents. Improved lipophilicity with reduced water solubility is an essential factor. Different online software are available for the computation of these factors, such as the Molinspiration property calculation toolkit, Osiris Property explorer, PASS prediction, etc. The drug-like characters, such as water solubility (clogS), the number of rotatable bonds (NROTB), hydrophobicity (clogP), molecular weight (MW), and the drug-like score of Lipinski’s rule of five [37,38,39], calculated for the lead compounds (**5a**–**r**) are represented in Table 3. Most of the synthesized compounds showed an acceptable range of solubility (clogS), which is less than (−4.6). The synthesized lead with a clogP-value < 5 is regarded as the compound with a better drug-likeness profile [40,41]. Most of the compounds were found to have a clogP-value of <5, which indicated their potential for oral administration. Parameters such as the TPSA (topological polar surface area) of new pyrazole derivatives (**5a**–**r**) were found to be within the acceptable range of 39.08–108.08 A°. The Molinspiration property calculation toolkit used for calculating the drug-likeness properties recommends that derivatives with negative or zero values should not be regarded as a drug-like candidates. However, all synthesized compounds, except **5c** and **5i**, were found to have scores > 0, as shown in Table 3. The predicted toxicity risks of all the synthesized derivatives were envisaged by Osiris Property Explorer and are given in Table 3. The compounds, except for **5c**, **5i**, and **5j**, were found to be non-mutagenic and non-carcinogenic. PASS prediction was performed for the synthesized compounds to omit the compounds with a probability of activity (Pa) < 0.400 [42,43]. The remaining compounds were selected for evaluation based on the net probability of activity over inactivity (Pa-Pi), being more than 0.400 (Table 3).

## 3. Materials and Methods

### 3.1. General Chemistry

The chemicals and reagents employed were of LR grade and procured from Sigma Aldrich (Mumbai, India), E. Merck (Mumbai, India), S.D. Fine Chemicals Ltd. (Mumbai, India), and Qualigens (New Delhi, India). Thin-layer chromatography (TLC) was carried out to observe the advancement of the reactions using benzene and acetone (80:20, *v*/*v*), as well as toluene/ethyl acetate/formic acid (50:40:10, *v*/*v*/*v*) as a solvent system, and spots were traced using iodine vapor or UV-light (254 nm). Melting points of the synthesized molecules were calculated using an electrical melting point apparatus (open capillary method) and are uncorrected. The infrared spectra were noted in the region from the 4000 to 400 cm^−1^ range on the Shimadzu FT-IR spectrometer, while ^1^H and ^13^C-NMR spectra were recorded on the Bruker Advance-500 MHz and 125 MHz spectrometer using DMSO-*d*_6_ or CDCl_3_ as an NMR solvent. Synapt-mass spectrometric detection was performed on UPLC-MS (Q-TOF-ESI; Waters Corp., Milford, MA, USA) using the ESI technique. Elemental analysis was executed on the CHNOS-Elemental analyzer (Vario EL III) and found to be ±0.4%, i.e., within the theoretical values.

#### 3.1.1. Synthesis of Substituted (*E*)-1-(1-(4-(Benzyloxy)phenyl)ethylidene)-2-phenylhydrazine (**3**)

A solution of **1** (10 mmol) and **2** (15 mmol) in absolute ethanol (30 mL) was refluxed for 6–8 h in the presence of glacial acetic acid (0.3 mL). The reaction advancement was determined by the TLC method using a mixture of ethyl acetate: hexane (8:2). The product obtained was then washed, dried in shade, and recrystallized from ethanol [44]; white solid, yield: 78%; m.p.: 123–125 °C.

#### 3.1.2. Synthesis of Substituted (*E*)-3-(4-(Benzyloxy)phenyl)-1-phenyl-1*H*-pyrazole-4-carbaldehyde (**4**)

Compound **3** in dry dimethylformamide at a temperature of 0–5 °C in a 100 mL RBF was reacted with POCl_3_ (drop-wise addition) for a time interval of 15–20 min and then heated in a steam bath for a period of 2 h; allowed to cool down to room temperature; poured in crushed ice; and then basified with a saturated solution of NaHCO_3_. The product obtained was then filtered, washed 2–3 times with water, dried, and recrystallized from ethanol to get yellow crystals (needle-like) [45].

White solid, yield: 72%; m.p.: 127–129 °C, IR (KBr, cm^−1^): 3093 (CH aromatic), 1710 (C=O), 1633 (C=N), and 1626 (C=C), 1255 (C-O). ^1^H NMR (CDCl_3_, 500 MHz) δ (ppm): 5.13 (s, 2H,-CH_2_-benzyloxy), 6.57 (d, 2H, Ar-H, *J* = 8.0 Hz), 7.05 (d, 2H, Ar-H, *J* = 8.4 Hz), 7.21 (d, 1H, Ar-H, *J* = 8.2 Hz), 7.30–7.59 (m, 4H, Ar-H), 7.67 (d, 2H, Ar-H, *J* = 8.8 Hz), 7.75–7.99 (m, 3H, Ar-H), 8.45 (s, 1H, pyrazole-CH), and 9.82 (s, 1H, CHO). Analysis calculated for C_23_H_18_N_2_O_2_: C, 77.93; H, 5.11; and N, 7.90. Found: C, 77.81; H, 5.16; and N, 7.81.

#### 3.1.3. Synthesis of (*E*)-3-(3-(4-(Benzyloxy)phenyl)-1-phenyl-1*H*-pyrazol-4-yl)-1-phenylprop-2-en-1-one derivatives (**5a**–**r**)

A mixture of 3-(4-(benzyloxy)phenyl)-1-phenyl-1*H*-pyrazole-4-carbaldehyde (**4**; 10 mmol) and different substituted acetophenones (10 mmol) were dissolved in 20 mL ethanol. To this mixture, sodium hydroxide (40%, 2 mL) was added at 0–5 °C and then stirred at room temperature for 24 h. Then, this reaction mixture was poured over crushed ice and acidified with dilute HCl (10%) until pH = 5. The light-yellow solid thus obtained was filtered, washed with water, dried, and recrystallized from ethanol to obtain the final product, i.e., (*E*)-3-(3-(4-(benzyloxy)phenyl)-1-phenyl-1*H*-pyrazol-4-yl)-1-phenylprop-2-en-1-one (**5a**–**r**) [46].

(*E*)-3-(3-(4-(benzyloxy)phenyl)-1-phenyl-1*H*-pyrazol-4-yl)-1-phenylprop-2-en-1-one (**5a**):

White solid, yield: 74%; m.p.: 190–194 °C; IR (KBr, cm^−1^): 3045 (CH aromatic), 2930 (CH aliphatic), 1710 (C=O), 1643 (C=C), 1627 (C=N), and 1245 (C-O); ^1^H NMR (CDCl_3_, 500 MHz); δ (ppm): 5.15 (s, 2H, CH_2_-benzyloxy), 7.12 (d, 2H, Ar-H, *J* = 8.4 Hz), 7.05 (d, 2H Ar-H, *J* = 8.4 Hz), 7.36 (t, 1H, Ar-H, *J* = 7.5 Hz), 7.54–7.62 (m, 5H, Ar-H), 7.64 (d, 2H, Ar-H, *J* = 8.8 Hz), 7.67 (d, 2H, Ar-H, *J* = 8.8 Hz), 7.74–7.85 (m, 3H, Ar-H), 7.89 (d, 2H, *J* = 7.7 Hz), 7.95 (d, 1H, chalcone H-7, *J =* 15.6 Hz), 8.01 (d, 1H, chalcone-H-6, *J =* 15.7 Hz), and 8.42 (s, 1H, pyrazole-H); ^13^C NMR (CDCl_3_, 125 MHz): δ (ppm) 71.1 (benzyloxy CH_2_), 113.1, 114.8, 119.4, 125.2, 126.7, 127.1, 127.7 (chalcone-C-7), 127.8, 128.4, 128.6, 129.1, 129.8, 130.2 (pyrazole-C-5), 134.5, 136.8, 137.9, 139.6 (pyrazole phenyl-C-1), 145.1 (chalcone-C-6), 150.7 (pyrazole-C-3), 159.1, and 189.8 (C=O); ESI-MS (*m*/*z*): 456.18 (M^+^). Analysis calculated for C_31_H_24_N_2_O_2_: C, 81.54; H, 5.29; N, 6.13; and O, 7.01. Found: C, 81.61; H, 5.22; and N, 6.17.

(*E*)-3-(3-(4-(benzyloxy)phenyl)-1-phenyl-1*H*-pyrazol-4-yl)-1-(2-chlorophenyl)prop-2-en-1-one (**5b**):

Off white solid, yield: 70%; m.p.: 182–184 °C; IR (KBr, cm^−1^): 3050 (CH aromatic), 2935 (CH aliphatic), 1715 (C=O), 1640 (C=N), 1597 (C=C), and 1255 (C-O), 745 (C-Cl); ^1^H NMR (CDCl_3_, 500 MHz); δ (ppm): 5.19 (s, 2H, CH_2_-benzyloxy), 6.97 (t, 2H, Ar-H, *J* = 8.7 Hz), 7.08 (d, 2H, Ar-H, *J* = 8.2 Hz), 7.31 (d, 2H, Ar-H, *J* = 9.6 Hz), 7.35 (t, 1H, Ar-H, *J* = 8.2 Hz), 7.57 (d, 2H, Ar-H, *J* = 8.3 Hz), 7.50–7.68 (m, 3H, Ar-H), 7.74 (d, 2H, Ar-H, *J* = 8.4 Hz), 7.96 (d, 2H, Ar-H, *J* = 7.6 Hz), 7.98 (d, 2H, Ar-H, *J* = 8.7 Hz), 7.93 (d, 1H, chalcone-H-7, *J =* 15.6 Hz), 8.06 (d, 1H, chalcone-H-6, *J =* 15.7 Hz), and 8.52 (s, 1H, pyrazole-H); and ^13^C NMR (CDCl_3_, 125 MHz): δ (ppm): 71.4 (benzyloxy-CH_2_), 113.8, 114.7, 119.5, 125.6, 126.4, 127.2, 127.8 (chalcone-C-7), 127.9, 128.4, 128.8, 129.5, 130.4 (pyrazole-C-5), 130.6, 131.8 (C-Cl), 136.2, 137.9, 139.4 (pyrazole-phenyl-C-1), 144.7 (Chalcone-C-6), 150.5 (pyrazole-C-3), 159.4, and 189.4 (C=O). ESI-MS (*m*/*z*): 491.88 (M^+^+1) and 492.75 (M^+^+2). Analysis calculated for C_31_H_23_ClN_2_O_2_: C, 75.82; H, 4.71; and N, 5.70. Found: C, 75.91; H, 4.62; and N, 5.75.

(*E*)-3-(3-(4-(benzyloxy)phenyl)-1-phenyl-1*H*-pyrazol-4-yl)-1-(3-nitrophenyl)prop-2-en-1-one (**5c**):

Orange red solid, yield: 78%; m.p.: 203–205 °C; IR (KBr, cm^−1^): 3046 (CH aromatic), 2940 (CH aliphatic), 1690 (C=O), 1625 (C=N), 1612 (C=C), 1510 and 1350 (NO_2_), and 1245 (C-O); ^1^H NMR (CDCl_3_, 500 MHz); δ (ppm): 5.11 (s, 2H, CH_2_-benzyloxy), 7.35 (d, 2H, Ar-H, *J* = 7.8 Hz), 7.44 (d, 2H, Ar-H, *J* = 7.8 Hz), 7.58 (t, 1H, Ar-H, *J* = 8.0 Hz), 7.63 (d, 2H, Ar-H, *J* = 8.8 Hz), 7.66–7.78 (m, 5H, Ar-H), 7.80 (d, 1H, chalcone-H-7, *J =* 15.5 Hz), 7.88 (d, 2H, Ar-H, *J* = 7.6 Hz), 8.00 (d, 1H, chalcone-H-6, *J =* 15.7 Hz), 8.02 (t, 1H, Ar-H, *J* = 8.8 Hz), 8.42 (s, 1H, Ar-H), 8.56 (d, 2H, Ar-H, *J* = 9.4 Hz), and 8.48 (s, 1H, pyrazole-H); ^13^C NMR (CDCl_3_, 125 MHz): δ (ppm) 71.2 (benzyloxy-CH_2_), 113.3, 114.5, 119.2, 123.7, 125.6, 126.6, 127.1, 127.6 (chalcone-C-7), 128.3, 128.8, 129.2, 129.4, 130.2 (pyrazole-C-5), 130.6, 134.9, 136.8, 138.5, 139.6 (pyrazole-phenyl-C-1), 143.3 (chalcone-C-6), 148.6 (Ar-C-NO_2_), 150.6 (pyrazole-C-3), 159.1, and 189.9 (C=O); ESI-MS (*m*/*z*): 501.17 (M^+^). Analysis calculated for C_31_H_23_N_3_O_4_: C, 74.21; H, 4.61; and N, 8.31. Found: C, 74.26; H, 4.55; and N, 8.42.

(*E*)-3-(3-(4-(benzyloxy)phenyl)-1-phenyl-1*H*-pyrazol-4-yl)-1-(3-hydroxyphenyl)prop-2-en-1-one (**5d**):

White buff solid, yield: 64%; m.p.: 184–186 °C; IR (KBr, cm^−1^): 3445 (OH), 3055 (CH aromatic), 2958 (CH aliphatic), 1705 (C=O), 1620 (C=N), 1597 (C=C), and 1245 (C-O); ^1^H NMR (CDCl_3_, 500 MHz); δ (ppm): 5.22 (s, 2H, CH_2_-benzyloxy), 5.42 (s, 1H, OH), 6.72 (d, 2H, Ar-H, *J* = 7.6 Hz), 6.94 (d, 2H, Ar-H, *J* = 7.8 Hz), 7.14 (s, 1H, Ar-H), 7.24 (t, 2H, Ar-H, *J* = 6.8 Hz), 7.31 (d, 2H, Ar-H, *J* = 6.4 Hz), 7.42–7.53 (m, 3H, Ar-H), 7.74 (d, 1H, chalcone-H-7, *J =* 15.8 Hz), 7.58–7.76 (m, 4H, Ar-pyrazole), 7.94 (d, 1H, chalcone-H-6, *J =* 16.0 Hz), 7.98 (d, 2H, Ar-H, *J* = 7.8 Hz), and 8.03 (s, 1H. pyrazole-H); ^13^C NMR (CDCl_3_, 125 MHz): δ (ppm) 70.8 (benzyloxy-CH_2_), 113.6, 114.7, 117.3, 119.4, 121.6, 123.2, 125.3, 126.3, 127.1, 127.4 (chalcone-C-7), 127.9, 128.3, 129.4, 130.5 (pyrazole-C-5), 131.4, 136.9, 137.3, 139.3 (pyrazole-phenyl-C-1), 146.4 (chalcone-C-6), 150.2 (pyrazole-C-3), 159.1, 164.8 (C-OH), and 196.1 (C=O); ESI-MS (*m*/*z*): 472.18 (M^+^). Analysis calculated for C_31_H_24_N_2_O_3_: C, 78.80; H, 5.13; and N, 5.94. Found: C, 78.85; H, 5.17; and N, 5.89

(*E*)-3-(3-(4-(benzyloxy)phenyl)-1-phenyl-1*H*-pyrazol-4-yl)-1-(4-chlorophenyl)prop-2-en-1-one (**5e**):

White buff solid, yield: 76%; m.p.: 212–214 °C; IR (KBr, cm^−1^): 3080 (CH aromatic), 2960 (CH aliphatic), 1720 (C=O), 1657 (C=C), 1610 (C=N), 1235 (C-O), and 790 (C-Cl); ^1^H NMR (CDCl_3_, 500 MHz); δ (ppm): 5.17 (s, 2H, CH_2_-benzyloxy), 7.15 (d, 2H, Ar-H, *J* = 8.7 Hz), 7.28 (d, 2H, Ar-H, *J* = 7.8 Hz), 7.31–7.43 (m, 3H, Ar-H), 7.46–7.55 (m, 4H, Ar-H), 7.59 (d, 2H, Ar-H, *J* = 8.4 Hz), 7.64 (d, 2H, Ar-H, *J* = 8.4 Hz), 7.83 (t, 3H, Ar-H, *J* = 7.8 Hz), 7.95 (d, 1H, chalcone-H-7, *J =* 15.6 Hz), 8.05 (d, 1H, chalcone-H-6), and 8.38 (s, 1H, pyrazole-H); ^13^C NMR (CDCl_3_, 125 MHz): δ (ppm) 69.7 (benzyloxy-CH_2_), 112.8, 115.9, 118.9, 119.2, 124.7, 126.1, 126.5, 128.5, 128.8 (chalcone-C-7), 128.9, 129.2, 129.9, 130.3 (pyrazole-C-5), 137.4, 139.9 (pyrazole-phenyl-C-1), 146.8 (chalcone-C-6), 150.7 (pyrazole-C-3), 158.6, and 189.7 (C=O); ESI-MS (*m*/*z*): 491.88 (M^+^+1) and 492.75 (M^+^+2). Analysis calculated for C_31_H_23_ ClN_2_O_2_: C, 75.82; H, 4.73, and N, 5.70. Found: C, 75.88; H, 4.69; and N, 5.75.

(*E*)-3-(3-(4-(benzyloxy)phenyl)-1-phenyl-1*H*-pyrazol-4-yl)-1-(4-fluorophenyl)prop-2-en-1-one (**5f**):

Off white solid, yield: 66%; m.p.: 168–170 °C; IR (KBr, cm^−1^): 3105 (CH aromatic), 2950 (CH aliphatic), 1708 (C=O), 1605 (C=N), 1593 (C=C), 1210 (C-O), and 755 (C-F); ^1^H NMR (CDCl_3_, 500 MHz); δ (ppm): 5.18 (s, 2H, CH_2_-benzyloxy), 7.16 (d, 2H, Ar-H, *J* = 8.7 Hz), 7.33 (t, 1H, Ar-H, *J* = 8.2 Hz), 7.43 (d, 2H Ar-H, *J* = 8.8 Hz), 7.54 (d, 2H, Ar-H, *J* = 8.2 Hz), 7.52–7.72 (m, 5H, Ar-H), 7.74 (d, 2H, Ar-H, *J* = 8.4 Hz), 7.76 (d, 1H, chalcone-H-7, *J =* 15.3 Hz), 7.96 (d, 2H, Ar-H, *J* = 7.6 Hz), 7.98 (d, 2H, Ar-H, *J* = 8.7 Hz), 8.06 (d, 1H, chalcone-H-6, *J =* 15.7 Hz), and 8.54 (s, 1H, pyrazole-H); ^13^C NMR (CDCl_3_, 125 MHz): δ (ppm) 70.7 (benzyloxy-CH_2_), 113.2, 114.5, 116.0, 119.6, 125.3, 126.4, 127.2, 127.4 (chalcone-C-7), 127.8, 128.6, 129.4, 130.4 (pyrazole-C-5), 131.8, 133.6, 136.1, 136.6, 139.7 (pyrazole-phenyl-C-1), 145.3 (chalcone-C-6), 150.8 (C-3-pyrazole), 159.6, 164.8 (C-F), and 189.5 (C=O); ESI-MS (*m*/*z*): 477.70 (M^+^+1). Analysis calculated for C_31_H_23_ FN_2_O_2_: C, 78.48; H, 4.90; and N, 5.92. Found: C, 78.55; H, 4.95; and N, 5.86.

(*E*)-3-(3-(4-(benzyloxy)phenyl)-1-phenyl-1*H*-pyrazol-4-yl)-1-(4-bromophenyl)prop-2-en-1-one (**5g**):

Off white solid, yield: 62%; m.p.: 178–180 °C; IR (KBr, cm^−1^): 3095 (CH aromatic), 2965 (CH aliphatic), 1708 (C=O), 1610 (C=N), 1585 (C=C), 1210 (C-O), and 665 (C-Br); ^1^H NMR (CDCl_3_, 500 MHz); δ (ppm): 5.14 (s, 2H, CH_2_-benzyloxy), 7.10–7.97 (m, 20H, aromatic H), 7.90 (d, 1H, CH=CH, Cis, *J* = 15.5 Hz), 7.83 (d, 1H, CH=CH, *J* = 9 Hz), and 8.33 (s, 1H, CH of pyrazole); ^13^C NMR (CDCl_3_, 125 MHz): δ (ppm) 70.1 (benzyloxy-CH_2_), 115.24, 118.01, 119.36, 120.56, 124.96, 125.86, 127.28, 127.52, 127.76, 128.10, 128.68 (chalcone-C-7), 129.61 (pyrazole-C-5), 129.92, 130.10 (pyrazole-phenyl-C-1), 131.90, 136.19 (chalcone-C-6), 136.79 (pyrazole-C-3), 139.40, 153.78 (C-Br), 159.36, and 188.92 (C=O); ESI-MS (*m*/*z*): 537.09 (M^+^+1). Analysis calculated for C_31_H_23_ BrN_2_O_2_: C, 69.55; H, 4.31; and N, 5.24. Found: C, 69.62; H, 4.36; and N, 5.20.

(*E*)-3-(3-(4-(benzyloxy)phenyl)-1-phenyl-1*H*-pyrazol-4-yl)-1-(4-hydroxyphenyl)prop-2-en-1-one (**5h**):

White buff solid, yield: 65%; m.p.: 167–169 °C; IR (KBr, cm^−1^): 3415 (OH), 3065 (CH aromatic), 2934 (CH aliphatic), 1695 (C=O), 1618 (C=N), 1605 (C=C), and 1248 (C-O); ^1^H NMR (CDCl_3_, 500 MHz); δ (ppm): 5.20 (s, 2H, CH_2_-benzyloxy), 5.68 (s, 1H, OH), 6.56 (d, 2H, Ar-H, *J* = 7.2 Hz), 6.80 (d, 2H, Ar-H, *J* = 7.6 Hz), 7.27 (t, 2H, Ar-H, *J* = 7.3 Hz), 7.31 (d, 2H, Ar-H, *J* = 6.4 Hz), 7.38–7.48 (m, 3H, Ar-H), 7.66 (d, 1H, chalcone-H-7, *J =* 15.5 Hz), 7.69–780 (m, 4H, Ar-pyrazole), 7.82 (d, 1H, chalcone-H-6, *J =* 15.5 Hz), 7.92 (d, 2H, Ar-H, *J* = 7.4 Hz), and 8.01 (s, 1H. pyrzole-H); ^13^C NMR (CDCl_3_, 125 MHz): δ (ppm) 71.8 (benzyloxy-CH_2_), 113.2, 114.5, 119.8, 125.8, 126.4, 127.2, 127.5 (chalcone-C-7), 127.9, 128.4, 128.8, 129.7, 130.5 (pyrazole-C-5), 130.8, 131.5, 136.8, 139.9 (pyrazole-phenyl-C-1), 146.4 (chalcone-C-6), 150.7 (pyrazole-C-3), 159.6, 164.5 (C-OH), and 189.8 (C=O); ESI-MS (*m*/*z*): 472.18 (M^+^). Analysis calculated for C_31_H_24_N_2_O_3_: C, 78.79; H, 5.12; and N, 5.93. Found: C, 78.84; H, 5.16; and N, 5.88.

(*E*)-3-(3-(4-(benzyloxy)phenyl)-1-phenyl-1*H*-pyrazol-4-yl)-1-(4-nitrophenyl)prop-2-en-1-one (**5i**):

Orange red solid, yield: 82%; m.p.: 223–225 °C IR (KBr, cm^−1^): 3035 (CH aromatic), 2956 (CH aliphatic), 1675 (C=O), 1630 (C=N), 1576 (C=C), 1505 and 1363 (NO_2_), and 1238 (C-O); ^1^H NMR (CDCl_3_, 500 MHz); δ (ppm): 5.12 (s, 2H, CH_2_-benzyloxy), 7.13 (d, 2H, Ar-H, *J* = 7.8 Hz), 7.42 (d, 2H, Ar-H, *J* = 8.6 Hz), 7.54 (t, 1H, Ar-H, *J* = 7.4 Hz), 7.62 (d, 2H, Ar-H, *J* = 8.8 Hz), 7.66–7.76 (m, 5H, Ar-H), 7.82 (d, 1H, chalcone-H-7, *J =* 15.6 Hz), 7.86 (d, 2H, Ar-H, *J* = 7.6 Hz), 8.08 (d, 1H, chalcone-H-6, *J =* 15.8 Hz), 8.16 (d, 2H, Ar-H, *J* = 8.6 Hz), 8.30 (d, 2H, Ar-H, *J* = 8.8 Hz), and 8.38 (s, 1H, pyrazole-H); ^13^C NMR (CDCl_3_, 125 MHz): δ (ppm) 69.5 (benzyloxy-CH_2_), 113.3 (pyrazole-C-4), 115.2, 119.6, 122.4, 125.6, 126.6, 127.5, 127.4 (chalcone-C-7), 127.8, 128.1, 128.6, 129.2, 130.8 (pyrazole-C-5), 130.6, 136.5, 139.6 (pyrazole-phenyl-C-1), 142.5 (chalcone-C-6), 150.5 (pyrazole-C-3), 153.6 (C-NO_2_), and 159.2, 189.3 (C=O); ESI-MS (*m*/*z*): 501.17 (M^+^). Analysis calculated for C_31_H_23_N_3_O_4_: C, 74.27; H, 4.64; and N, 8.41. Found: C, 74.32; H, 4.58; and N, 8.46.

(*E*)-3-(3-(4-(benzyloxy)phenyl)-1-phenyl-1*H*-pyrazol-4-yl)-1-(p-tolyl)prop-2-en-1-one (**5j**):

Off white solid, yield: 78%; m.p.: 205–207 °C; IR (KBr cm^−1^): 3028 (CH aromatic), 2960 (C-H str in CH_3_), 2968 (CH aliphatic), 1724 (C=O), 1645 (C=N), 1618 (C=C), and 1205 (C-O); ^1^H NMR (CDCl_3_, 500 MHz); δ (ppm): 3.75 (s, 3H, CH_3_), 5.16 (s, 2H, CH_2_-benzyloxy), 7.14 (d, 2H, Ar-H, *J* = 7.1 Hz), 7.29 (d, 2H, Ar-H, *J* = 5.1 Hz), 7.54 (t, 3H, Ar-H, *J* = 8.1 Hz), 7.62 (d, 2H, Ar-H, *J* = 8.4 Hz), 7.64–7.72 (m, 3H, Ar-H), 7.74 (d, 1H, chalcone-H-7, *J =* 15.4 Hz), 7.76–7.81 (m, 4H, Ar-H), 7.86 (d, 1H, chalcone-H-6, *J =* 15.1 Hz), 7.95 (d, 2H, Ar-H, *J* = 7.5 Hz), and 8.52 (s, 1H. pyrazole-H); ^13^C NMR (CDCl_3_, 125 MHz): δ (ppm)20.5 (Ar-CH_3_), 70.8 (benzyloxy-CH_2_), 11.9 (pyrazole-C-4), 115.3, 118.3, 119.2, 124.9, 126.1, 126.4, 128.2 (chalcone-C-7), 128.3, 128.9, 129.1, 129.7, 130.0 (pyrazole-C-5), 139.4 (pyrazol-phenyl-C-1), 146.8 (chalcone-C-6), 150.7 (pyrazole-C-3), 158.6, and 189.7 (C=O); ESI-MS (*m*/*z*): 471.20 (M^+^+1). Analysis calculated for C_32_H_26_N_2_O_2_: C, 81.67; H, 5.59; and N, 5.96. Found: C, 81.73; H, 5.53; and N, 6.02.

(*E*)-3-(3-(4-(benzyloxy)phenyl)-1-phenyl-1*H*-pyrazol-4-yl)-1-(4-methoxyphenyl)prop-2-en-1-one (**5k**):

Greenish solid, yield: 68%; m.p.: 187–189 °C; IR (KBr, cm^−1^): 3090 (CH aromatic), 2970 (CH aliphatic), 1745 (C=O), 1655 (C=N), 1633 (C=C), and 1267 (OCH_3_), 1220 (C-O); ^1^H NMR (CDCl_3_, 500 MHz); δ (ppm): 3.86 (s, 3H, OCH_3_), 5.14 (s, 2H, CH_2_-benzyloxy), 7.10–7.97 (m, 20H, aromatic H), 7.90 (d, 1H, CH=CH Cis, *J* = 8.5 Hz), 7.83 (d, 1H, CH=CH, *J* = 8 Hz), and 8.53 (s, 1H, CH of pyrazole); ^13^C NMR (CDCl_3_, 125 MHz): δ (ppm) 70.1 (Ar-OCH_3_), 70.5 (benzyloxy-CH_2_), 115.15 (pyrazole-C-4), 119.34, 119.74, 122.36 (chalcone-C-7), 124.10, 127.53, 127.93, 128.13, 128.68 (pyrazole-C-5), 129.70, 130.70, 131.20 (phenyl-pyrazole-C-1), 136.70 (chalcone-C-6), 139.06 (pyrazole-C-3), 154.51, 159.75, and 185.24 (C=O); ESI-MS (*m*/*z*): 487.22 (M^+^+1). Analysis calculated for C_32_H_26_N_2_O_3_: C, 78.97; H, 5.40; and N, 5.78. Found: C, 78.94; H, 5.34; and N, 5.84.

(*E*)-3-(3-(4-(benzyloxy)phenyl)-1-phenyl-1*H*-pyrazol-4-yl)-1-(3,4-dichlorophenyl)prop-2-en-1-one (**5l**):

Off white solid, yield: 76%; m.p.: 243–245 °C; IR (KBr, cm^−1^): 3030 (CH aromatic), 2980 (CH aliphatic), 1745 (C=O), 1653 (C=N), 1637 (C=C), 1225 (C-O), and 785 (C-Cl); ^1^H NMR (CDCl_3_, 500 MHz); δ (ppm): 5.17 (s, 2H, CH_2_-benzyloxy), 7.16 (d, 2H, Ar-H, *J* = 8.4 Hz), 7.25 (s, 1H Ar-H), 7.36 (d, 2H, Ar-H, *J* = 7.6 Hz), 7.48–7.51 (d, 2x 2H, Ar-H, *J =* 8.4 Hz), 7.55 (s, 1H, Ar-H), 7.56 (s, 1H, Ar-H), 7.58 (s, 1H, Ar-H), 7.67 (d, 2H, Ar-H, *J* = 8.4 Hz), 7.79–7.83 (m, 3H, Ar-H), 7.95 (d, 1H, chalcone-H, *J =* 15.6 Hz), 8.05 (d, 1H, chalcone-H, *J =* 16.2 Hz), and 8.37 (s, 1H, pyrazole-H); ^13^C NMR (CDCl_3_, 125 MHz): δ (ppm) 70.2 (benzyloxy-CH_2_), 113.4, 114.4, 119.6, 125.5, 126.2, 127.2, 127.4 (chalcone-C-7), 127.7, 128.3, 128.6, 128.8, 129.5, 130.2 (pyrazole-C-5), 130.6, 131.4, 133.6, 136.6, 137.3, 139.3 (C-Cl), 139.8 (phenyl-C-1), 145.2 (chalcone-C-6), 150.3 (pyrazole-C-3), 159.1, and 189.5 (C=O); ESI-MS (*m*/*z*): 524.11 (M^+^+1), 525.08 (M^+^+2), and 526.06 (M^+^+4). Analysis calculated for C_31_H_22_ Cl_2_N_2_O_2_: C, 70.85; H, 4.23; and N, 5.31. Found: C, 70.92; H, 4.18; and N, 5.41.

(*E*)-3-(3-(4-(benzyloxy)phenyl)-1-phenyl-1*H*-pyrazol-4-yl)-1-(2,4dichlorophenyl)prop-2-en-1-one (**5m**):

Off white solid, yield: 67%; m.p.: 192–194 °C; IR (KBr, cm^−1^): 3065 (CH aromatic), 2985 (CH aliphatic), 1753 (C=O), 1620 (C=N), 1598 (C=C), 1275 (C-O), and 785 (C-Cl); ^1^H NMR (CDCl_3_, 500 MHz); δ (ppm): 5.12 (s, 2H, CH_2_-benzyloxy), 7.14 (d, 2H, Ar-H, *J* = 8.4 Hz), 7.53 (d, 2H, Ar-H, *J* = 8.4 Hz), 7.46–7.52 (d, 2x 2H, Ar-H, *J =* 8.4 Hz), 7.57 (s, 1H, Ar-H), 7.55 (s, 1H, Ar-H), 7.59 (d, 2H, Ar-H, *J* = 7.8 Hz), 7.72 (d, 2H, Ar-H, *J* = 8.4 Hz), 7.75–7.80 (m, 3H, Ar-H), 7.62 (d, 1H, chalcone-H, *J =* 15.5 Hz), 7.69 (d, 1H, chalcone-H, 15.3 Hz), and 8.47 (s, 1H, pyrazole-H); ^13^C NMR (CDCl_3_, 125 MHz): δ (ppm) 70.6 (benzyloxy-CH_2_), 113.3, 114.6, 119.7, 125.4, 126.5, 127.2, 127.3 (chalcone-C-7), 127.6, 128.7, 129.2, 129.3, 129.7, 130.3 (pyrazole-C-5), 131.7 (C-Cl), 133.3, 135.4, 136.9, 139.7 (phenyl-C-1), 145.3 (chalcone-C-6), 146.3, 150.2 (pyrazole-C-7), 159.2, and 189.1 (C=O); ESI-MS (*m*/*z*): 524.11 (M^+^+1), 525.08 (M^+^+2), and 526.06 (M^+^+4). Analysis calculated for C_31_H_22_ Cl_2_N_2_O_2_: C, 70.85; H, 4.21; and N, 5.32. Found: C, 70.92; H, 4.18; and N, 5.41.

(*E*)-3-(3-(4-(benzyloxy)phenyl)-1-phenyl-1*H*-pyrazol-4-yl)-1-(2,4-dihydroxyphenyl)prop-2-en-1-one (**5n**):

White buff solid, yield: 72%; m.p.: 176–178 °C; IR (KBr, cm^−1^): 3435 (OH), 3086 (CH aromatic), 2950 (CH aliphatic), 1782 (C=O), 1620 (C=N), 1603 (C=C), and 1250 (C-O); ^1^H NMR (CDCl_3_, 500 MHz); δ (ppm): 5.18 (s, 2H, CH_2_-benzyloxy), 5.60 (s, 2H, 2x OH), 6.42 (s, 1H, Ar-H), 7.10 (t, 3H, Ar-H, *J* = 6.8 Hz), 7.38 (d, 2H, Ar-H, *J* = 6.4 Hz), 7.46–7.64 (m, 3H, Ar-H), 7.72 (d, 1H, chalcone-H-7, *J =* 15.2 Hz), 7.62–7.76 (m, 4H, Ar-pyrazole), 7.80 (d, 1H, chalcone-H-6, *J =* 15.8 Hz), 7.86 (d, 2H, Ar-H, *J* = 8.8 Hz), 7.90 (d, 2H, Ar-H, *J* = 7.6 Hz), and 8.05 (s, 1H. pyrazole-H); ^13^C NMR (CDCl_3_, 125 MHz): δ (ppm) 71.2 (benzyloxy-CH_2_), 104.6, 113.2, 113.5, 114.3, 114.7, 119.6, 125.4, 126.4, 127.8, 127.4 (chalcone-C-7), 127.5, 128.5, 128.6, 129.4, 130.4 (pyrazole-C-5), 133.5, 136.4, 139.8 (pyrazole-phenyl-C-1), 145.2 (chalcone-C-6), 150.6 (pyrazole-C-3), 159.2, 165.4 (C’-4-OH), 166.8 (C’-2-OH), and 192.2 (C=O); ESI-MS (*m*/*z*): 488.17 (M^+^). Analysis calculated for C_31_H_24_N_2_O_4_: C, 76.21; H, 4.95; and N, 5.73. Found: C, 76.28; H, 4.88; and N, 5.76.

(*E*)-3-(3-(4-(benzyloxy)phenyl)-1-phenyl-1*H*-pyrazol-4-yl)-1-(3,4-dimethoxyphenyl)prop-2-en-1-one (**5o**):

Green solid, yield: 79%; m.p.: 206–208 °C; IR (KBr, cm^−1^): 3085 (CH aromatic), 2955 (CH aliphatic), 1730 (C=O), 1610 (C=N), 1596 (C=C), 1225 (OCH_3_), and 1265 (C-O); ^1^H NMR (CDCl_3_, 500 MHz); δ (ppm): 3.96 (s, 6H, OCH_3_), 5.12 (s, 2H, CH_2_-benzyloxy), 6.91–7.97 (m, 19H, aromatic H), 7.90 (d, 1H, CH=CH, Cis, *J* = 13 Hz), 7.7 (d, 1H, CH=CH, *J* = 9 Hz), and 8.32 (s, 1H, CH of pyrazole); ^13^C NMR (CDCl_3_, 125 MHz): δ (ppm) 56.1 (C’-4-OCH_3_), 70.1 (benzyloxy-CH_2_), 109.9 (Pyrazole-C-4), 110.7, 115.2, 118.27, 119.33, 120.93, 122.79, 125.21, 126.83, 127.15, 127.52, 128.09, 128.68, 129.59 (Chalcone-C-7), 130.13, 131.36, 134.75 (pyrazole-C-5), 136.80, 139.50 (pyrazole-phenyl-C-1), 149.18 (chalcone-C-6), 151.15 (pyrazole-C-3), 153.51, 159.28 (C-4′-OCH_3_), and 188.25 (C=O); ESI-MS (*m*/*z*): 517.19 (M^+^). Analysis calculated for C_33_H_28_N_2_O_4_: C, 76.73; H, 5.46; and N, 5.42. Found: C, 76.78; H, 5.40; and N, 5.48.

(*E*)-3-(3-(4-(benzyloxy)phenyl)-1-phenyl-1*H*-pyrazol-4-yl)-1-(2,4-dimethoxyphenyl)prop-2-en-1-one (**5p**):

Green solid, yield: 76%; m.p.: 226–228 °C; IR (KBr, cm-1): 3096 (CH aromatic), 2967 (CH aliphatic), 1738 (C=O), 1625 (C=N), 1563 (C=C), 1245 (OCH_3_), and 1272 (C-O); ^1^H NMR (CDCl_3_, 500 MHz); δ (ppm): 3.73–3.75 (s, 6H, 2xOCH_3_), 5.17 (s, 2H, CH_2_-benzyloxy), 7.14 (d, 2H, Ar-H, *J* = 8.6 Hz), 7.35 (d, 2H, Ar-H, *J* = 8.1 Hz), 7.38 (s, 1H, Ar-H), 7.39–7.60 (m, 10H, Ar-H), 7.67 (d, 2H, Ar-H, *J* = 8.7 Hz), 7.99 (d, 1H, chalcone-H-7, *J* = 15.3 Hz), 8.36 (s, 1H, chalcone-H-6), and 8.54 (s, 1H. pyrazole-H); ^13^C NMR (CDCl_3_, 125 MHz): δ (ppm) 56.3 (C’-4-OCH_3_), 69.5 (benzyloxy-CH_2_), 113.4 (pyrazole-C-4), 113.7, 114.2, 119.6, 119.1, 125.6, 126.3, 127.3, 127.65 (chalcone-C-7), 127.2, 128.3, 128.5, 129.4, 130.4 (pyrazole-C-5), 130.8, 133.7, 136.4, 139.3 (pyrazole-phenyl-C-1), 145.5 (chalcone-C-6), 150.7 (pyrazole-C-3), 151.2 (C-3′-OCH_3_), 156.2 (C-4′-OCH_3_), 159.5, and 189.5 (C=O); ESI-MS (*m*/*z*): 516.20 (M^+^). Analysis calculated for C_33_H_28_N_2_O_4_: C, 76.73; H, 5.46; and N, 5.42. Found: C, 76.75; H, 5.38; and N, 5.45.

(*E*)-3-(3-(4-(benzyloxy)phenyl)-1-phenyl-1*H*-pyrazol-4-yl)-1-(2-bromo-4-methoxyphenyl)prop-2-en-1-one (**5q**):

Off white solid, yield: 56%; m.p.: 174–176 °C; IR (KBr, cm^−1^): 3070 (CH aromatic), 2938 (CH aliphatic), 1765 (C=O), 1647 (C=N), 1588 (C=C), 1229 (C-OCH_3_), 1260 (C-O), and 630 (C-Br); ^1^H NMR (CDCl_3_, 500 MHz); δ (ppm): 3.82 (s, 3H, OCH_3_), 5.13 (s, 2H, CH_2_-benzyloxy), 7.14 (d, 2H, Ar-H, *J* = 7.1 Hz), 7.21 (s, 1H, Ar-H), 7.38 (d, 2H, Ar-H, *J* = 6.4 Hz), 7.32–7.44 (m, 5H, Ar-H), 7.48 (s, 1H, Ar-H), 7.62 (d, 2H, Ar-pyrazole, *J* = 7.6 Hz), 7.64 (d, 2H, Ar-H, *J* = 7.6 Hz), 7.86 (d, 1H, chalcone-H-7, *J =* 15.5 Hz), 7.90 (d, 2H, Ar-H, *J* = 7.6 Hz), 8.32 (d, 1H, chalcone-H-6, *J =* 16.4 Hz), and 8.43 (s, 1H. pyrazole-H); ^13^C NMR (CDCl_3_, 125 MHz): δ (ppm) 56.3 (C’-4-OCH_3_), 69.5 (benzyloxy-CH_2_), 113.4 (pyrazole-C-4), 113.7, 114.2, 119.6, 119.1, 125.6, 126.3, 127.3, 127.65 (chalcone-C-7), 127.2, 128.3, 128.5, 129.4, 130.4 (pyrazole-C-5), 130.8, 133.7, 136.4, 139.3 (pyrazole-phenyl-C-1), 145.5 (chalcone-C-6), 150.7 (pyrazole-C-3), 151.2 (C-3′-OCH_3_), 156.2 (C-4′-OCH_3_), 159.5, and 189.5 (C=O); ESI-MS (*m*/*z*): 525.17 (M^+^). Analysis calculated for C_32_H_25_ BrN_2_O_3_: C, 67.97; H, 4.46; amd N, 4.95. Found: C, 68.02; H, 4.42; and N, 4.98.

(*E*)-3-(3-(4-(benzyloxy)phenyl)-1-phenyl-1*H*-pyrazol-4-yl)-1-(4-(trifluoromethyl)Phenyl)prop-2-en-1-one (**5r**):

White buff solid, yield: 68%; m.p.: 186–188 °C; IR (KBr, cm^−1^): 3075 (CH aromatic), 2985 (CH aliphatic), 1760 (C=O), 1650 (C=N), 1520 (C=C), and 1265 (C-O), 780 (C-F); ^1^H NMR (CDCl_3_, 500 MHz); δ (ppm): 5.14 (s, 2H, CH_2_-benzyloxy), 7.17 (d, 2H, Ar-H, *J* = 7.2 Hz), 7.36 (t, 1H, Ar-H, *J* = 7.3 Hz), 7.44–7.52 (m, 5H, Ar-H), 7.58 (d, 2H, Ar-H, *J* = 7.5 Hz), 7.69 (d, 2H Ar-H, *J* = 8.4 Hz), 7.74 (d, 2H, Ar-H, *J* = 8.4 Hz), 7.80 (d, 2H, Ar-H, *J* = 8.7 Hz), 7.88 (d, 1H, chalcone-H-7, *J =* 15.6 Hz), 7.92 (d, 2H, Ar-H, *J* = 8.6 Hz), 8.06 (d, 1H, chalcone-H-6, *J* = 15.76 Hz), and 8.34 (s, 1H, pyrazole-H); ^13^C NMR (CDCl_3_, 125 MHz): δ (ppm) 70.6 (benzyloxy-CH_2_), 113.1, 114.9, 119.8, 124.5 (C-F_3_), 125.4, 125.7, 126.2, 127.1, 127.4 (chalcone-C-7), 127.9, 128.5, 128.8, 129.4, 130.2 (C-2′), 130.3 (pyrazole-C-5), 136.4, 136.93 (C-4′), 139.8 (pyrazole-phenyl-C-1), 141.4, 145.7 (chalcone-C-6), 150.6 (pyrazole-C-3), 159.1, and 189.6 (C=O); ESI-MS (*m*/*z*): 554.17 (M^+^+1). Analysis calculated for C_32_H_23_ F_3_N_2_O_2_: C, 73.27; H, 4.42; and N, 5.34. Found: C, 73.36; H, 4.36; and N, 5.42.

### 3.2. Molecular Docking Simulation

The molecular docking study of the designed molecules was carried out to assess their interaction and binding modes with the target receptors using Glide Extra precision (XP) Maestro 10.1 Schrodinger running on the Linux 64 operating system [47]. The 2D structure of the synthesized compounds was generated and then converted to their respective 3D structures using Ligprep. The PDB file of the X-ray crystal structure of the tubulin domain bound to colchicine (PDB ID:3E22) was downloaded from the RCSB Protein Data Bank. The protein preparation wizard was used to prepare the protein and the grid was generated for the co-crystal ligand using receptor grid generation. The water residues beyond 5 Å were eliminated. The protein was optimized by assigning H-bonds and the minimization of the OPLS 2005 force field. The docked pose of ligands and their interactions were analyzed after the end of the molecular docking.

### 3.3. Biological Activity

#### 3.3.1. In Vitro Anticancer Activity by MTT Assay

##### Materials and Methods

(3-(4,5-Dimethyl-2-yl)-2,5-diphenyl tetrazolium bromide (MTT), Dulbecco’s modified Eagle’s medium (DMEM), 0.25% trypsin, and 0.02% EDTA mixture were purchased from HiMedia (Mumbai, India), and fetal bovine serum (FBS) was obtained from Gibco (Grand Island, NY, USA).

##### Cell Line and Culture Conditions

Human cervical cancer cell line SiHa, human breast cancer cell line MCF-7, human prostate cancer cell line PC-3, and human embryonic kidney HEK-293 cells were procured from the National Centre for Cell Sciences (NCCS) Pune, India. In this experiment, antibiotics (100 units/mL penicillin and 100 mg L^−1^ streptomycin were grown as a monolayer culture in Dulbecco’s modified Eagle’s medium containing 10% fetal bovine serum) were cultured in a humidified atmosphere of 5% CO_2_ at 37 °C in T-75 flasks and subcultured twice a week.

##### In Vitro Cytotoxicity

The cytotoxic effects of the selected compounds were evaluated by MTT assay [48] on the above-mentioned cell lines. Initially, 2 × 10^4^ cells/well were seeded into 96-well plates (150 µL/well) in triplicates and allowed to grow. The cells were incubated for 24 h and subsequently treated with varying concentrations of the compounds. After 48 h, the cells were incubated with 20 μL of MTT (5 mg/mL in PBS) in a fresh medium for 4 h at 37 °C. MTT is a metabolic substrate that is reduced to give formazan crystals, which were solubilized in DMSO (150 µL/well) and analyzed by reading the absorbance at 540 nm after 15 min of the incubation period on the iMark Microplate Reader (Bio-Rad). Percentage viability and IC_50_ values were used to determine the relative absorbance of treated versus control (untreated) cells.

#### 3.3.2. In Vitro Tubulin Polymerization Assay

Tubulin polymerization is a dynamic process by enhancement of fluorescence intensity due to the combination of a fluorescent reporter into the microtubules as polymerization occurs [49]. Tubulin polymerization was performed by using a purified brain tubulin polymerization kit purchased from Cytoskeleton (BK110P, Denver, CO, USA). The tubulin polymerization assay was monitored by the increase in fluorescence over a 60 min period at 37 °C, with excitation at 360 nm and emission at 450 nm. The final buffer concentration for tubulin polymerization contained 80 mM PIPES, pH 6.9; 2 mM MgCl_2_; 0.5 mM EGTA; 1μM GTP; and 15% glycerol. Firstly, 5 µL of the test compounds (final concentration of 10 µM) was added and then warmed to 37 °C for 1 min. The reaction was initiated by adding 50 µL of the tubulin reaction mix as specified for 6 min.

#### 3.3.3. In Silico Bioactivity Study

All newly synthesized derivatives were screened for their physicochemical properties, oral bioavailability, toxicity, and online biological activity by using online software such as Molinspiration, Osiris Property Explorer, and the PASS prediction study. Hydrophobicity (c-log P), molecular weight (MW), number of rotatable bonds (NROTB) of Lipinski’s rule of five [33], water solubility (c-log S), toxicity [35], and drug-likeness [34] were calculated using the online Molinspiration property calculation toolkit and online OSIRIS Property explorer. With the help of Osiris Property Explorer software, the toxicities of the synthesized derivatives were predicted, which showed that the newly synthesized compounds would be free of carcinogenicity, mutagenicity, reproductive adverse effects, and irritation. All these derivatives were also projected for their pharmacological activity by using the online PASS computer program (prediction of activity spectra for substances).

### 3.4. Statistical Analysis

Statistical analyses were performed using Graph Pad Prism 5 software. All data were analyzed by ANOVA, followed by Dunnett’s multiple comparison test for *n* = 6; (a) *p* < 0.05 and (b) *p* < 0.001. Relative to normal and data were analyzed by paired Student’s *t*-test for *n* = 6; (c) *p* < 0.0001 and (d) *p* < 0.005.

## 4. Conclusions

A series of *E*-3-(3-(4-(benzyloxy)phenyl)-1-phenyl-1*H*-pyrazol-4-yl)-1-phenylprop-2-en-1-one conjugates were synthesized, characterized, and evaluated for anticancer potential and tubulin polymerization inhibition. The conjugates **5d**, **5k**, **5n**, **5o**, and **5p** revealed potent cytotoxic activities against MCF-7 (human breast), SiHa (human cervical), PC-3 (human prostate), and non-cancerous cell lines using combretastatin A4 as the standard. Compound **5o** displayed the most potent cytotoxic activity with the IC_50_ value of 2.13 ± 0.80 μM for the MCF-7 cancer cell line. Furthermore, compound **5o** considerably arrested the cell cycle, induced apoptosis in a dose-dependent manner, and inhibited polymerization of tubulin by 66.40%. Molecular docking studies of compound **5o** (highest docking score of −7.22) showed that the colchicine-binding site of tubulin established promising interactions with ASN 249, ALA 250, LYS 254, SER 178, and TYR 224, and pi-cation interaction with LYS 352 in the active site of tubulin. The in silico bioactivity study and PASS prediction studies exposed that most of the synthesized compounds displayed excellent physicochemical properties within the ideal range. These results established that compound 5o is a newer tubulin polymerization inhibitor and is commendable of advanced investigation in the future, directing to the progress of newer potential anticancer agents. Therefore, these conjugates can further be structurally modified to promote a new potential target for the optimization and development of anticancer agents.

## Figures and Tables

**Table 1:**
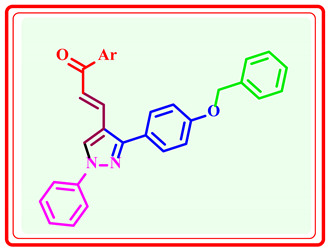
Docking score and anti-tubulin activity of *E*-3-(3-(4-(benzyloxy) phenyl)-1-phenyl-1*H*-pyrazol-4-yl)-1-phenylprop-2-en-1-one analogs (**5a**–**r**).

Comp.	Ar	Docking Score	% Inhibition of Tubulin Polymerization at 10 µM ^b^	Inhibition of Tubulin Polymerization IC_50_ (μM) ^a^
**5a**	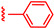	−5.994	Nd	Nd
**5b**	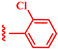	−5.934	Nd	Nd
**5c**	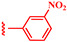	−5.873	Nd	Nd
**5d**	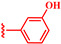	−6.967	49.78	1.65 ± 0.06
**5e**	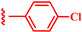	−6.937	48.44	1.58 ± 0.04
**5f**	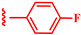	−6.241	Nd	Nd
**5g**	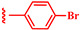	−5.531	Nd	Nd
**5h**	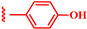	−5.752	Nd	Nd
**5i**	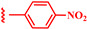	−6.249	28.42	3.58 ± 0.83
**5j**	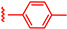	−5.952	Nd	Nd
**5k**	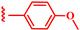	−6.432	32.79	3.03 ± 0.44
**5l**	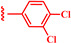	−7.072	55.17	1.73 ± 0.04
**5m**	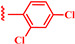	−6.664	33.76	2.97 ± 0.03
**5n**	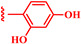	−6.751	47.56	2.73 ± 0.07
**5o**	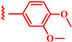	−7.002	66.40	1.15 ± 0.06
**5p**	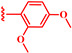	−7.277	51.11	1.93 ± 0.03
**5q**	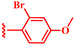	−6.111	Nd	Nd
**5r**	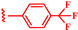	−6.367	31.50	2.34 ± 0.63
Co-crystal–colchicine	----	−7.059	----	----
Control	----	---	0.0	0.0
Paclitaxel (3 µM)	----	---	−25.73	0.53 ± 0.12
Combretastatin A-4 (6 µM)	----	---	72.30	1.46 ± 0.05
Vincristine (3 µM)	----	---	75.45	1.54 ± 0.54

^a^ Half-maximal inhibitory concentration: compound concentration required to inhibit tubulin polymerization by 50%; data are the mean ± SD of *n* = 3 independent experiments performed in triplicates. ^b^ Inhibition of tubulin polymerization at 10 μM (final volume = 10 mL); compounds were pre-incubated with tubulin at a final concentration of 10 μΜ.

**Table 2:** In vitro cytotoxic effect (IC_50_, μM) of the *E*-3-(3-(4-(benzyloxy) phenyl)-1-phenyl-1*H*-pyrazol-4-yl)-1-phenylprop-2-en-1-one against a panel of human cancer and normal cell lines.

Comp.	Cell Line (IC_50_) ^a^			
	MCF-7 ^b^	SiHa ^c^	PC3 ^d^	HEK-293 ^e^
**5d**	4.10 ± 1.12	4.85 ± 0.89	2.97 ± 0.88	>50
**5e**	11.50 ± 1.62	12.3 ± 1.32	9.68 ± 1.42	>50
**5i**	42.70 ± 0.34	34.53 ± 0.90	13.14 ± 1.6	45.23 ± 2.60
**5k**	7.23 ± 0.74	5.65 ± 0.71	5.45 ± 0.94	>50
**5l**	8.23 ± 1.45	11.70 ± 2.83	5.84 ± 1.65	>50
**5m**	17.14 ± 2.25	6.46 ± 1.52	11.22 ± 1.44	>50
**5n**	6.98 ±1.12	3.60 ± 0.45	3.56 ± 0.63	>50
**5o**	2.13 ± 0.80	4.34 ± 0.98	4.46 ± 0.53	>50
**5p**	3.45 ± 1.28	4.98 ± 0.54	6.52 ± 1.23	>50
**5r**	14.82 ± 2.76	>50	>50	38.3 ± 1.72
**CA-4**	4.12 ± 0.38	5.23 ± 0.85	3.86 ± 0.39	19.25 ± 1.65

Cell lines were treated with different concentrations of compounds for 48 h as described under experimental protocol. Cell viability was measured by employing MTT assay. ^a^ IC_50_ values are the concentrations causing 50% inhibition of cancer cell growth (µM). Data represent the mean values ± standard deviation of three independent experiments performed in triplicates. ^b^ Breast cancer, ^c^ cervical cancer, ^d^ prostate cancer cell line, and ^e^ human normal cell line CA-4: Combretastatin A-4.

**Table 3:** In silico physicochemical properties for oral bioavailability and bioactivity of the test compounds using computational predictive software.

Comp.	Cancer Treatment		cLogP	cLogS	*n* OHNH	*n* ON	MW	Rotatable Bonds	Drug Likeness	Drug Score	TPSA	Toxicity
	Pa	Pa-Pi										
**5a**	0.65	0.64	4.59	−4.60	0	4	456.18	8	0.97	0.34	61.80	NM, NC
**5b**	0.40	0.30	3.20	−2.23	1	4	491.88	8	1.23	0.24	63.08	NM. NC
**5c**	0.25	0.08	4.20	−3.42	1	4	501.17	8	−2.34	0.26	39.08	SR
**5d**	0.43	0.34	4.34	−3.36	0	4	472.18	8	3.45	0.35	65.08	NM, NC
**5e**	0.40	0.29	4.80	−2.07	1	4	491.88	8	4.62	0.05	68.08	NM, NC
**5f**	0.37	0.25	3.40	−4.34	0	4	477.70	8	2.82	0.36	63.60	NM, NC
**5g**	0.47	0.46	3.60	−3.45	0	4	535.09	8	1.24	0.32	70.08	NM, NC
**5h**	0.43	0.42	4.56	−2.67	1	4	472.18	8	2.87	0.23	62.07	NM, NC
**5i**	0.35	0.23	4.90	−3.60	1	4	501.17	8	−2.24	0.25	80.08	SM
**5j**	0.49	0.493	4.90	−3.78	1	4	471.20	8	1.37	0.11	102.30	SM
**5k**	0.44	0.36	2.34	−3.56	0	10	487.19	10	−1.92	0.34	87.00	NM, NC
**5l**	0.43	0.42	2.23	−4.02	1	7	524.11	9	3.45	0.25	62.90	NM, NC
**5m**	0.23	0.22	4.25	−3.35	2	5	524.11	8	2.64	0.15	59.31	NM, NC
**5n**	0.49	0.41	3.60	−2.54	1	5	488.17	9	−1.56	0.56	63.31	NM, NC
**5o**	0.47	0.40	3.45	−2.63	1	6	516.20	10	1.67	0.34	63.56	NM, NC
**5p**	0.48	0.41	2.50	−2.67	1	4	516.20	8	2.12	0.27	63.34	NM, NC
**5q**	0.29	0.13	5.20	−3.24	3	7	525.17	9	3.44	0.48	80.20	NM, NC
**5r**	0.31	0.16	4.50	−3.48	3	7	554.17	9	2.65	0.65	75.23	NM, NC

Pa: probability of being active; Pi: the probability of being inactive; cLogP: lipophilicity; cLogS: water solubility; *n* OHNH: number of hydrogen bond donors; *n* ON: number of hydrogen bond acceptors; MW: molecular weight; TPSA; topological polar surface area; NM: non-mutagenic; NC: non-carcinogenic; SM: slightly mutagenic; and SR: slightly reproducible.

## Data Availability

Data is contained within the article and supplementary material.

## Supplementary Materials

The following are available online at https://www.mdpi.com/article/10.3390/ph15030280/s1, Figure S1: ^1^H NMR of (*E*)-3-(3-(4-(benzyloxy)phenyl)-1-phenyl-1*H*-pyrazol-4-yl)-1-(3,4-dimethoxyphenyl)prop-2-en-1-one; Figure S2: ^13^C NMR of (*E*)-3-(3-(4-(benzyloxy)phenyl)-1-phenyl-1*H*-pyrazol-4-yl)-1-(3,4-dimethoxyphenyl)prop-2-en-1-one; Figure S3: ^1^H NMR of (*E*)-3-(3-(4-(benzyloxy)phenyl)-1-phenyl-1*H*-pyrazol-4-yl)-1-(3,4-dimethoxyphenyl)prop-2-en-1-one; Figure S4: ^13^C NMR of (*E*)-3-(3-(4-(benzyloxy)phenyl)-1-phenyl-1*H*-pyrazol-4-yl)-1-(3,4-dimethoxyphenyl)prop-2-en-1-one; Figure S5: Mass spectra of (*E*)-3-(3-(4-(benzyloxy)phenyl)-1-phenyl-1*H*-pyrazol-4-yl)-1-(3,4-dimethoxyphenyl)prop-2-en-1-one; Figure S6: ^1^H NMR of (*E*)-3-(3-(4-(benzyloxy)phenyl)-1-phenyl-1*H*-pyrazol-4-yl)-1-(4-bromophenyl)prop-2-en-1-one; Figure S7: ^1^H NMR of (*E*)-3-(3-(4-(benzyloxy)phenyl)-1-phenyl-1*H*-pyrazol-4-yl)-1-(4-bromophenyl)prop-2-en-1-one; Figure S8: ^13^C NMR of (*E*)-3-(3-(4-(benzyloxy)phenyl)-1-phenyl-1*H*-pyrazol-4-yl)-1-(4-bromophenyl)prop-2-en-1-one; Figure S9: Mass spectra of (*E*)-3-(3-(4-(benzyloxy)phenyl)-1-phenyl-1*H*-pyrazol-4-yl)-1-(4-bromophenyl)prop-2-en-1-one; Figure S10: ^1^H NMR of (*E*)-3-(3-(4-(benzyloxy)phenyl)-1-phenyl-1*H*-pyrazol-4-yl)-1-(4-methoxyphenyl)prop-2-en-1-one; Figure S11: ^13^C NMR of (*E*)-3-(3-(4-(benzyloxy)phenyl)-1-phenyl-1*H*-pyrazol-4-yl)-1-(4-methoxyphenyl)prop-2-en-1-one; Figure S12: ^1^H NMR of (*E*)-3-(3-(4-(benzyloxy)phenyl)-1-phenyl-1*H*-pyrazol-4-yl)-1-(4-methoxyphenyl)prop-2-en-1-one; Figure S13: ^13^C NMR of (*E*)-3-(3-(4-(benzyloxy)phenyl)-1-phenyl-1*H*-pyrazol-4-yl)-1-(4-methoxyphenyl)prop-2-en-1-one; Figure S14: Mass spectra of (*E*)-3-(3-(4-(benzyloxy)phenyl)-1-phenyl-1*H*-pyrazol-4-yl)-1-(4-methoxyphenyl)prop-2-en-1-one; and Figure S15: Effect of lead conjugates **5d**, **5e**, **5i**, **5k**, **5l**, **5m**, **5n**, **5o**, **5p**, and **5r** on tubulin polymerization. Tubulin polymerization was monitored by the increase in fluorescence at 360 nm (excitation) and 420 nm (emission) for 1 h at 37 °C. Combretastatin A-4 was used as the reference standard in this study.
