# Supplementary material for: Design, Synthesis, Molecular Docking, and Biological Evaluation of Pyrazole Hybrid Chalcone Conjugates as Potential Anticancer Agents and Tubulin Polymerization Inhibitors
Source: Pharmaceuticals (Basel). 2022 Feb 24;15(3):280. doi: 10.3390/ph15030280 (PMC8954831; doi:10.3390/ph15030280)
Supplement: Supplementary file 1 [file pharmaceuticals-15-00280-s001.zip › pharmaceuticals-1555145-supplementary.pdf]

Design, Synthesis, Molecular Docking, and Biological Evaluation of Pyrazole Hybrid Chalcone Conjugates as Potential Anticancer Agents and Tubulin Polymerization Inhibitors

Md. Jahangir Alam[1], Ozair Alam[1,*], Ahmad Perwez[2], Moshahid Alam Rizvi[2], Mohd Javed Naim[1], V.G.M. Naidu[3], Mohd Imran[4], Mohammed M. Ghoneim[5], Sultan Alshehri[6] and Faiyaz Shakeel[6]

[1] Medicinal Chemistry and Molecular Modeling Lab, Department of Pharmaceutical Chemistry, School of Pharmaceutical Education and Research, Jamia Hamdard, New Delhi 110062, India; zahangir.jh@gmail.com (M.J.A.), oalam@jamiahamdard.ac.in (O.A), javednaim88@rediffmail.com (M.J.N.)

[2] Department of Biochemistry and Molecular Biology, Faculty of Bioscience, Jamia Millia Islamia, New Delhi 110020, India; ahmad86.delhi@gmail.com (A.P.), rizvi_ma@yahoo.com (M.A.R.)

[3] National Institute of Pharmaceutical Education and Research, Guwahati 781101, Assam, India; vgmnaidu@gmail.com

[4] Department of Pharmaceutical Chemistry, Faculty of Pharmacy, Northern Border University, Rafha 91911, Saudi Arabia; imran.pchem@gmail.com

[5] Department of Pharmacy Practice, College of Pharmacy, AlMaarefa University, Ad Diriyah 13713, Saudi Arabia; mghoneim@mcst.edu.sa

[6] Department of Pharmaceutics, College of Pharmacy, King Saud University, Riyadh 11451, Saudi Arabia; salshehri1@ksu.edu.sa (S.A.), fsahmad@ksu.esu.sa (F.S.)

* Correspondence: oalam@jamiahamdard.ac.in

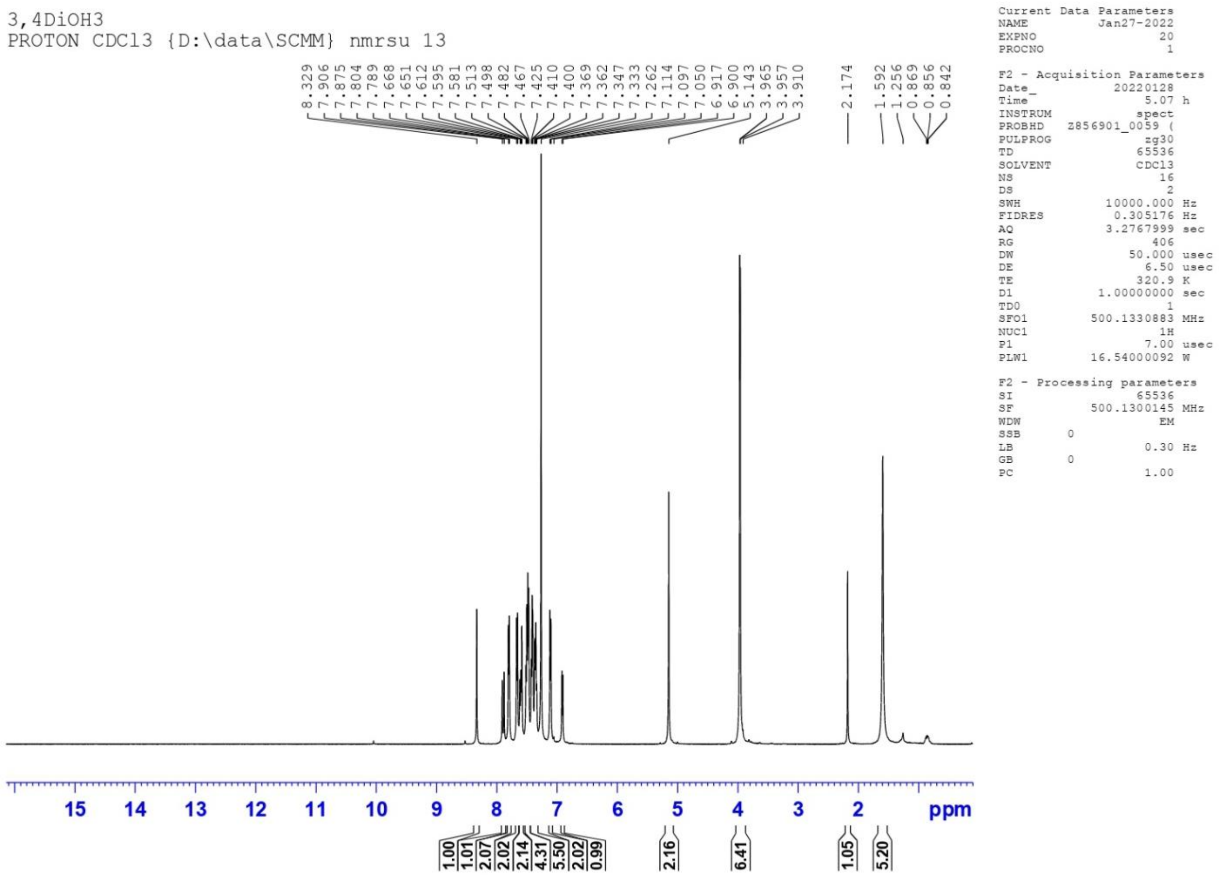

Figure S1. ^{1}H NMR of (E)-3-(3-(4-(benzyloxy)phenyl)-1-phenyl-1H-pyrazol-4-yl)-1-(3,4-dimethoxyphenyl)prop-2-en-1-one.

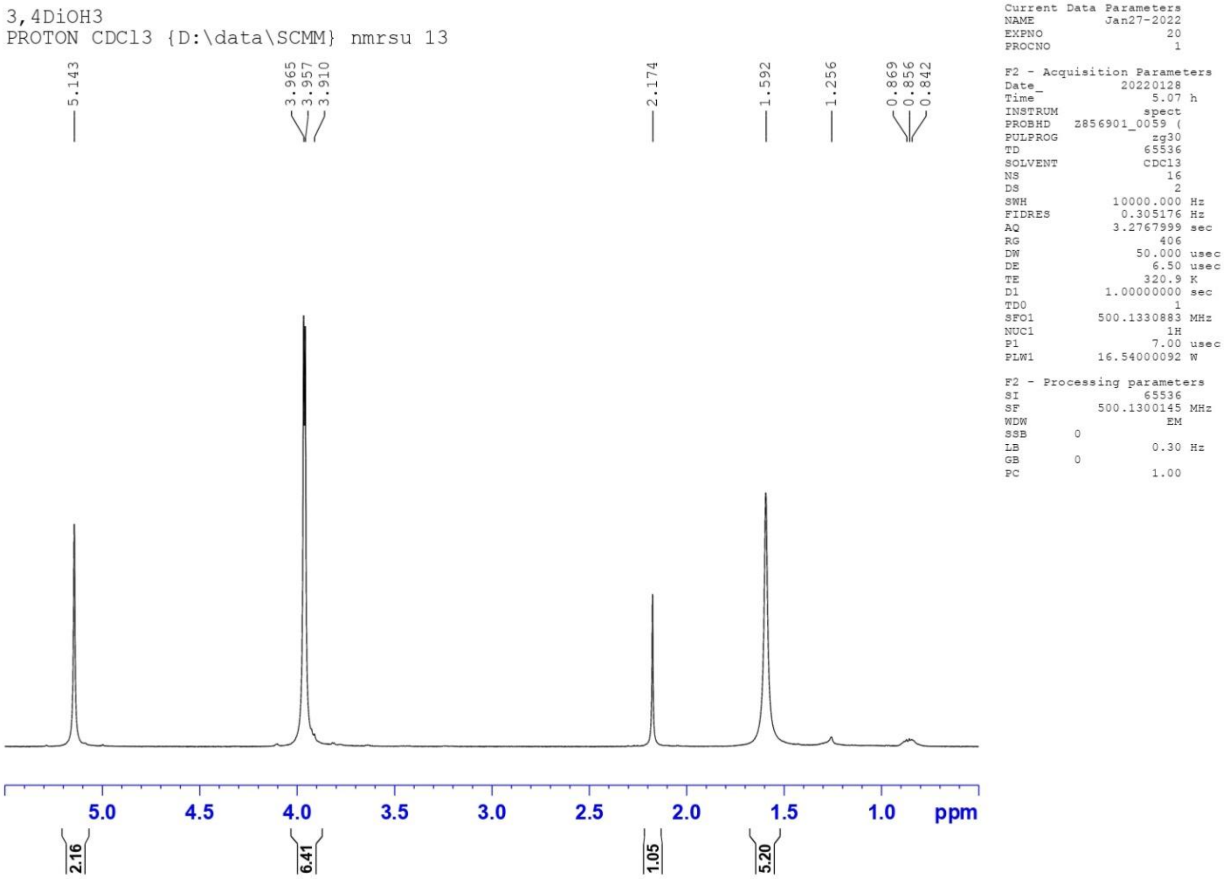

Figure S2. ^{13}C NMR of (E)-3-(3-(4-(benzyloxy)phenyl)-1-phenyl-1H-pyrazol-4-yl)-1-(3,4-dimethoxyphenyl)prop-2-en-1-one.

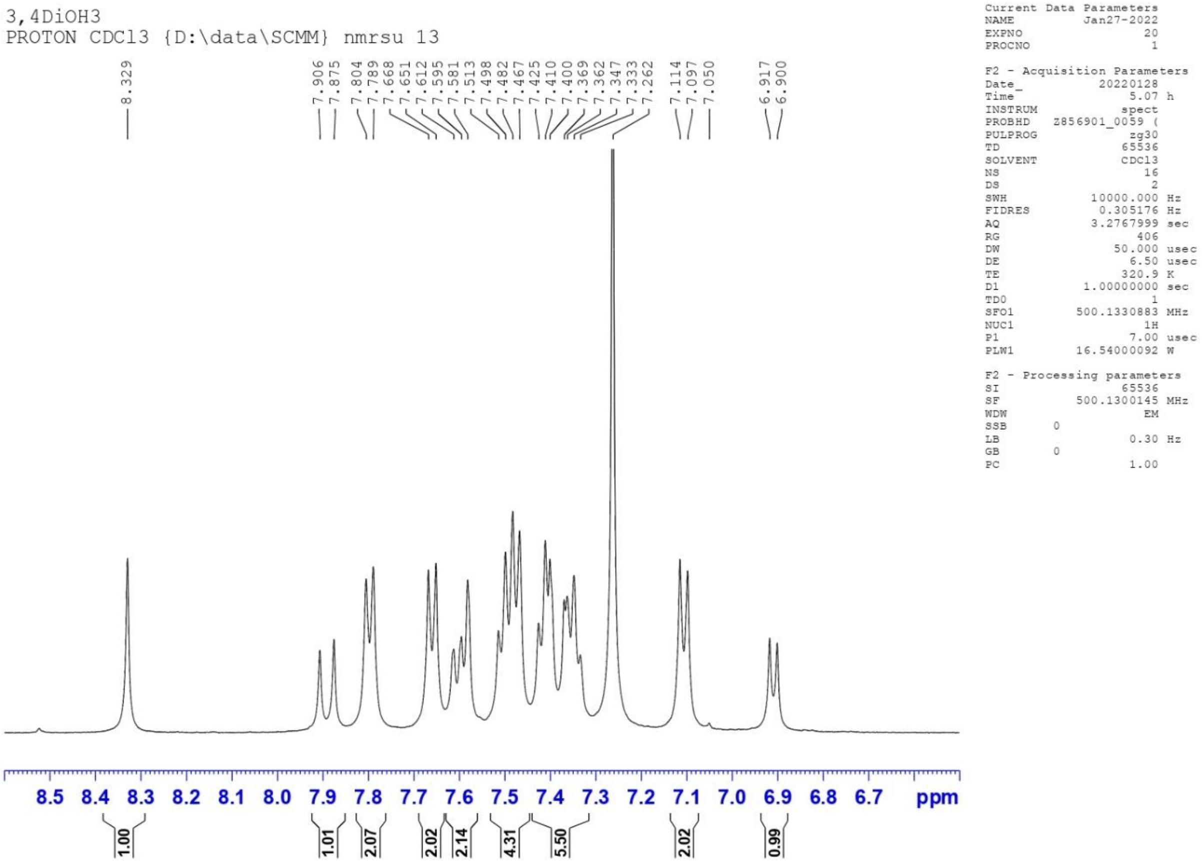

Figure S3. ^{1}H NMR of (E)-3-(3-(4-(benzyloxy)phenyl)-1-phenyl-1H-pyrazol-4-yl)-1-(3,4-dimethoxyphenyl)prop-2-en-1-one.

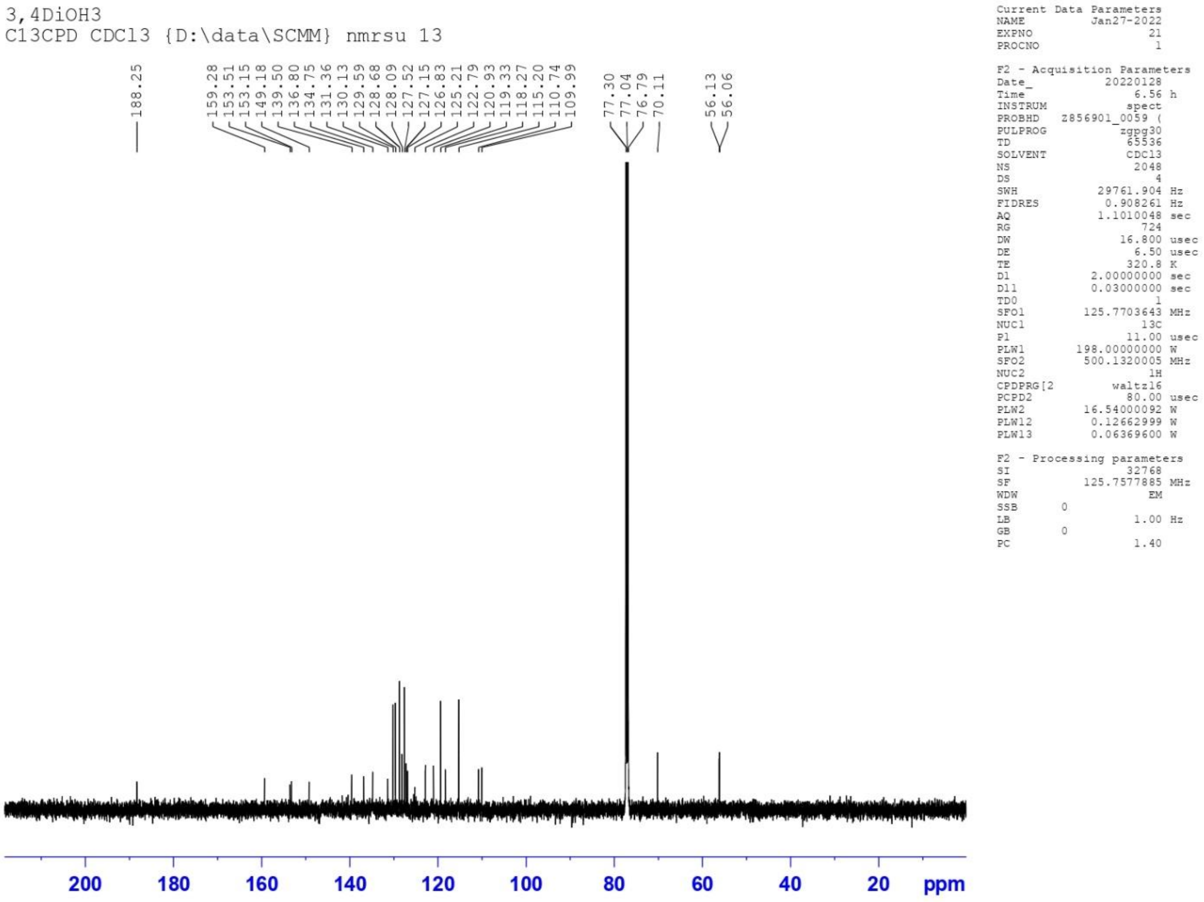

Figure S4. ^{13}C NMR of (E)-3-(3-(4-(benzyloxy)phenyl)-1-phenyl-1H-pyrazol-4-yl)-1-(3,4-dimethoxyphenyl)prop-2-en-1-one.

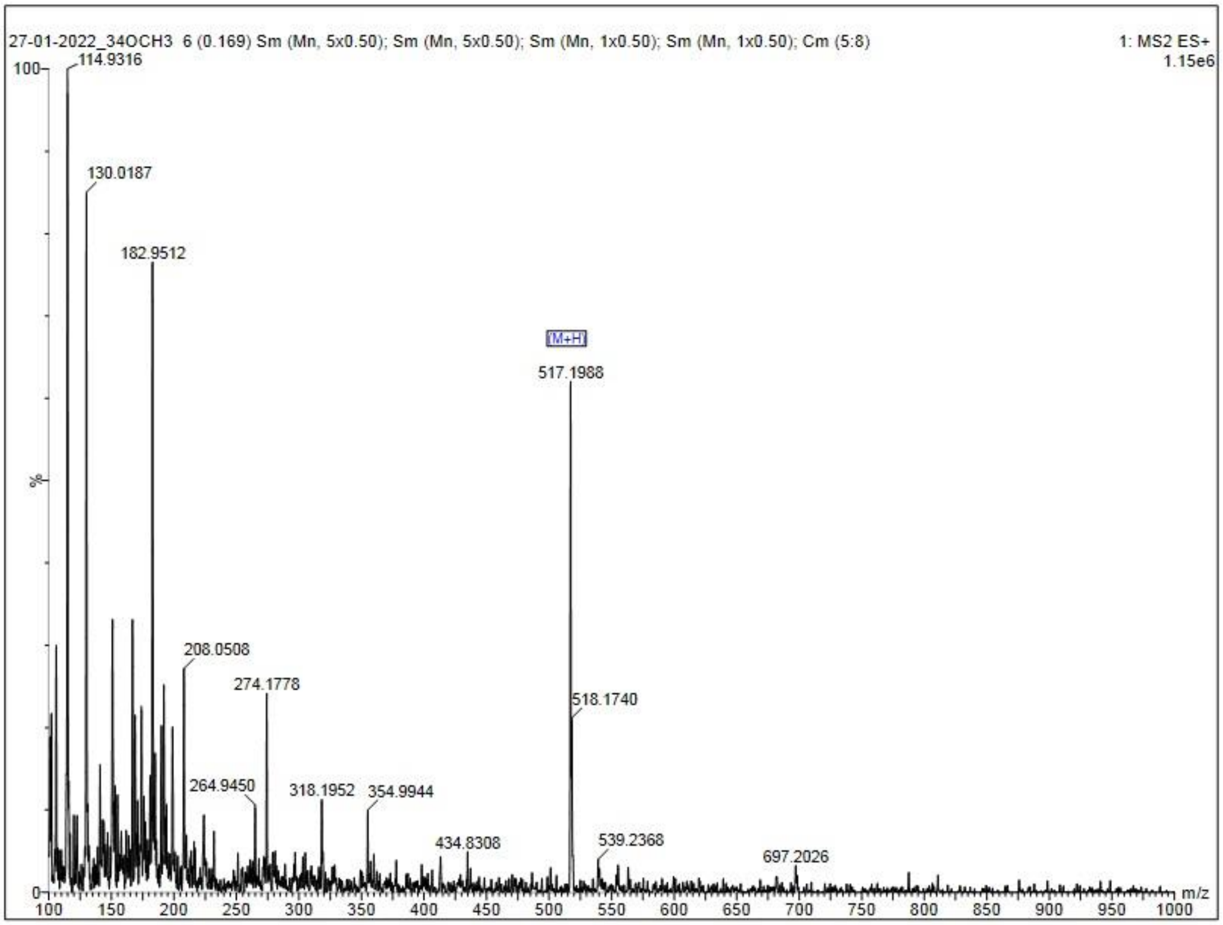

Figure S5. Mass spectra of (E)-3-(3-(4-(benzyloxy)phenyl)-1-phenyl-1H-pyrazol-4-yl)-1-(3,4-dimethoxyphenyl)prop-2-en-1-one.

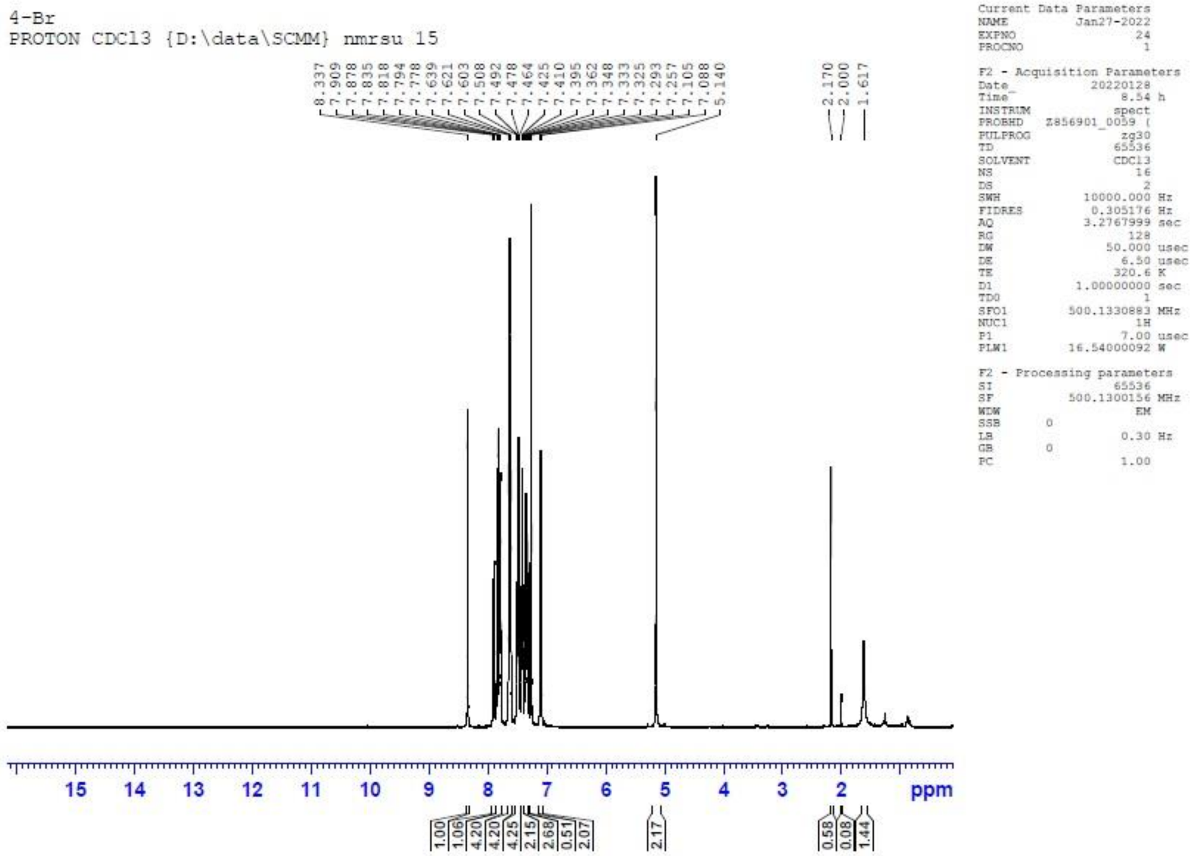

Figure S6. ^{1}H NMR of (E)-3-(3-(4-(benzyloxy)phenyl)-1-phenyl-1H-pyrazol-4-yl)-1-(4-bromophenyl)prop-2-en-1-one.

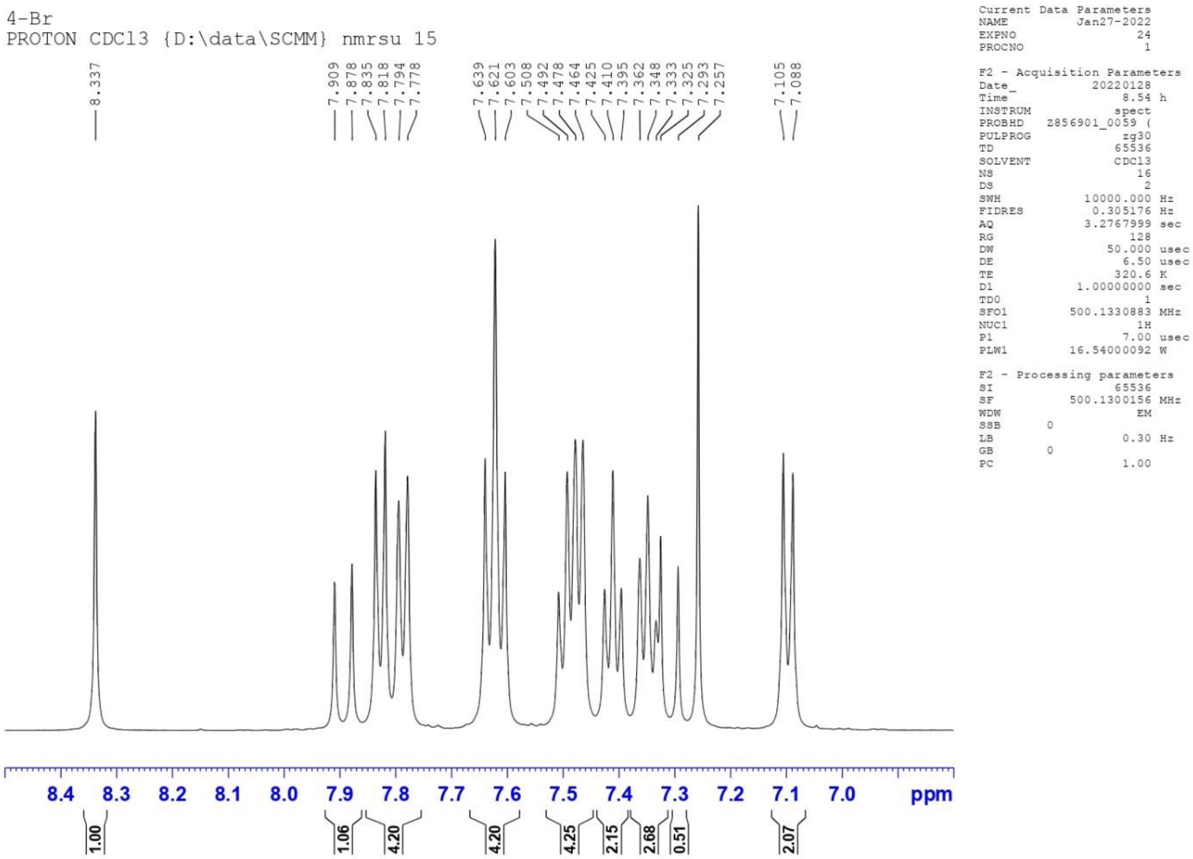

Figure S7. ^{1}H NMR of (E)-3-(3-(4-(benzyloxy)phenyl)-1-phenyl-1H-pyrazol-4-yl)-1-(4-bromophenyl)prop-2-en-1-one.

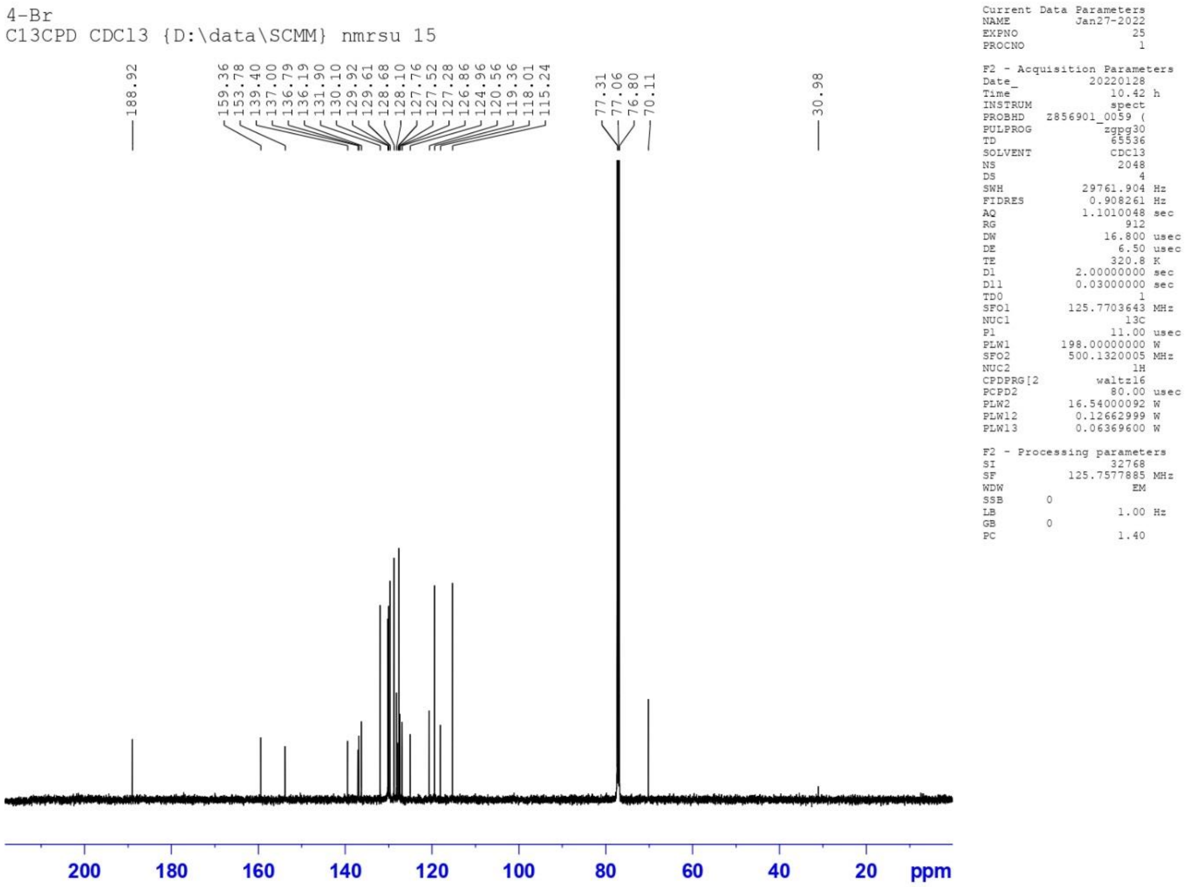

Figure S8. ^{13}C NMR of (E)-3-(3-(4-(benzyloxy)phenyl)-1-phenyl-1H-pyrazol-4-yl)-1-(4-bromophenyl)prop-2-en-1-one.

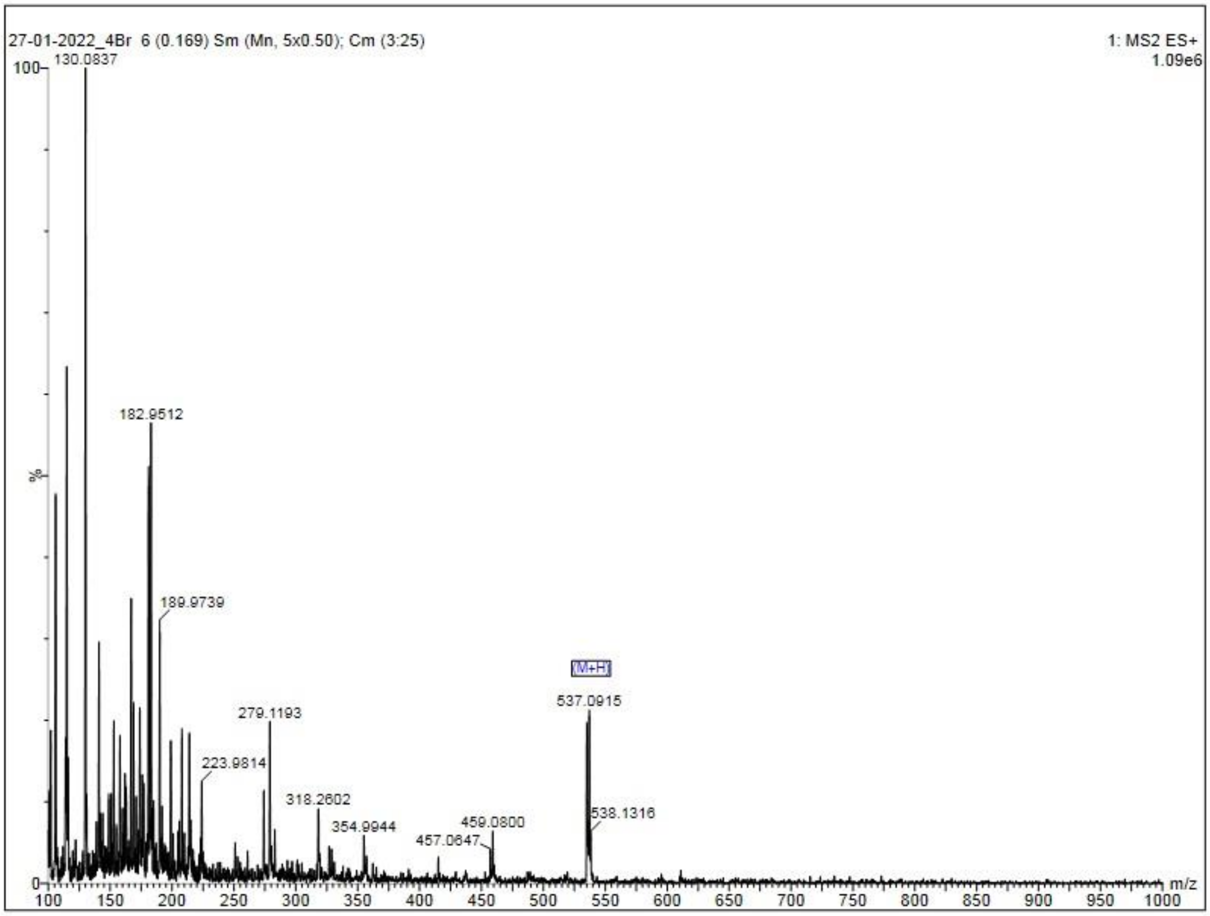

Figure S9. Mass spectra of (E)-3-(3-(4-(benzyloxy)phenyl)-1-phenyl-1H-pyrazol-4-yl)-1-(4-bromophenyl)prop-2-en-1-one.

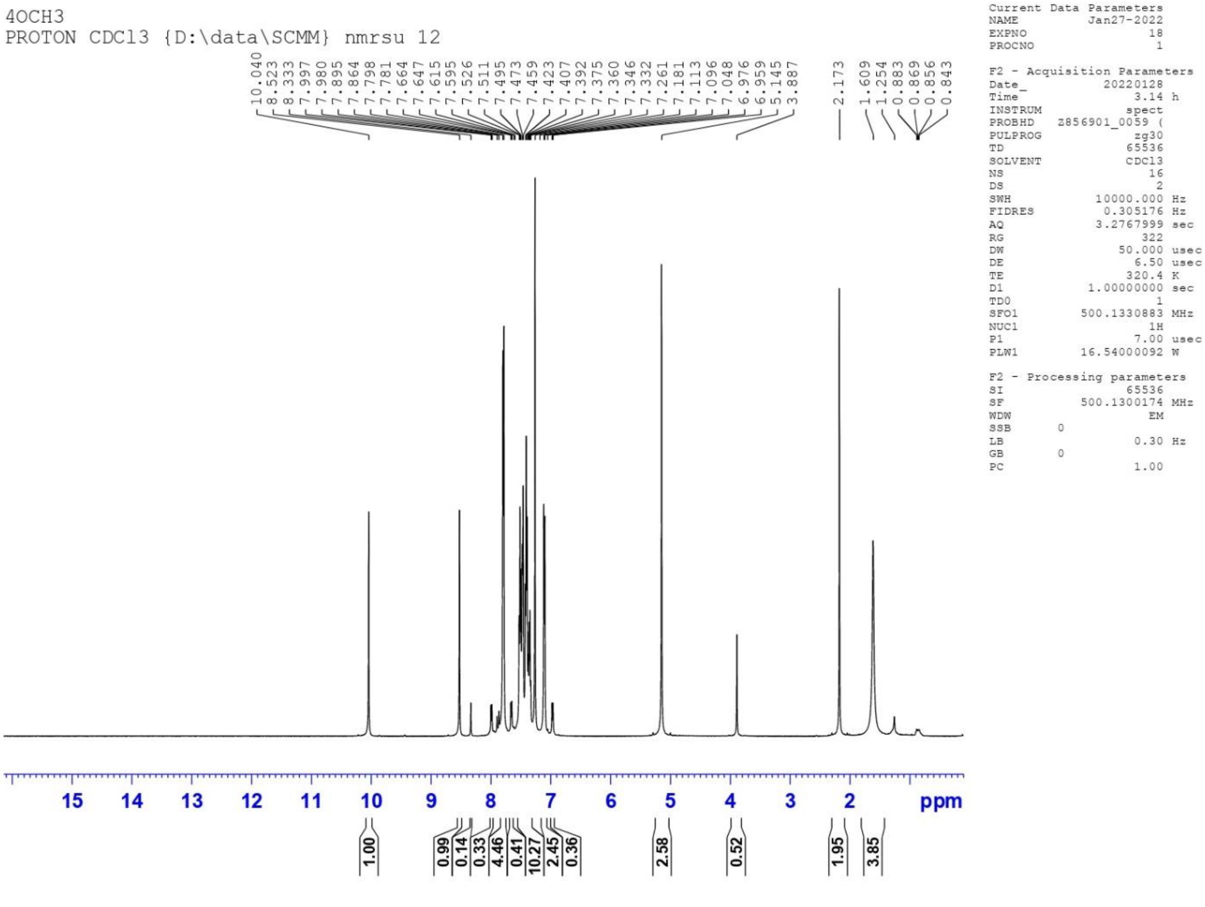

Figure S10. ^{1}H NMR of (E)-3-(3-(4-(benzyloxy)phenyl)-1-phenyl-1H-pyrazol-4-yl)-1-(4-methoxyphenyl)prop-2-en-1-one.

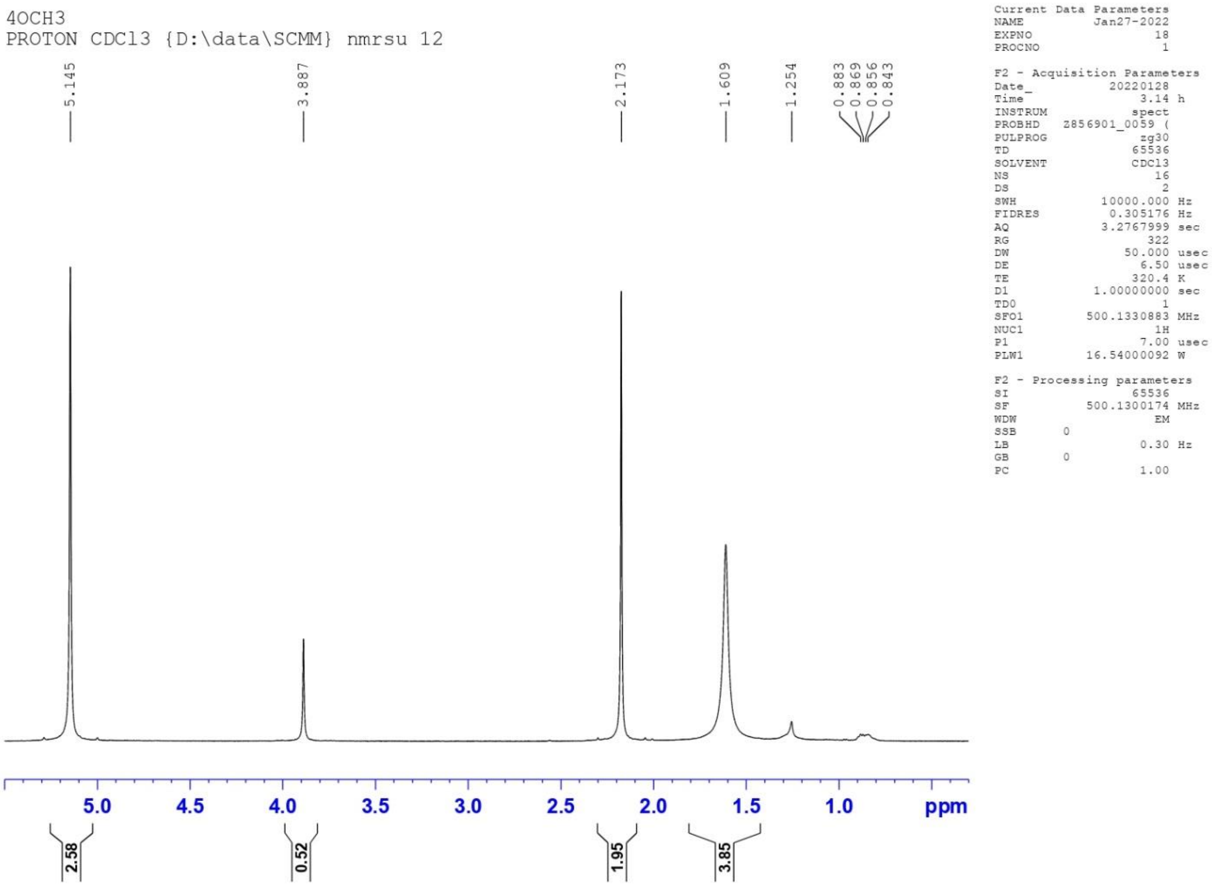

Figure S11. ^{13}C NMR of (E)-3-(3-(4-(benzyloxy)phenyl)-1-phenyl-1H-pyrazol-4-yl)-1-(4-methoxyphenyl)prop-2-en-1-one.

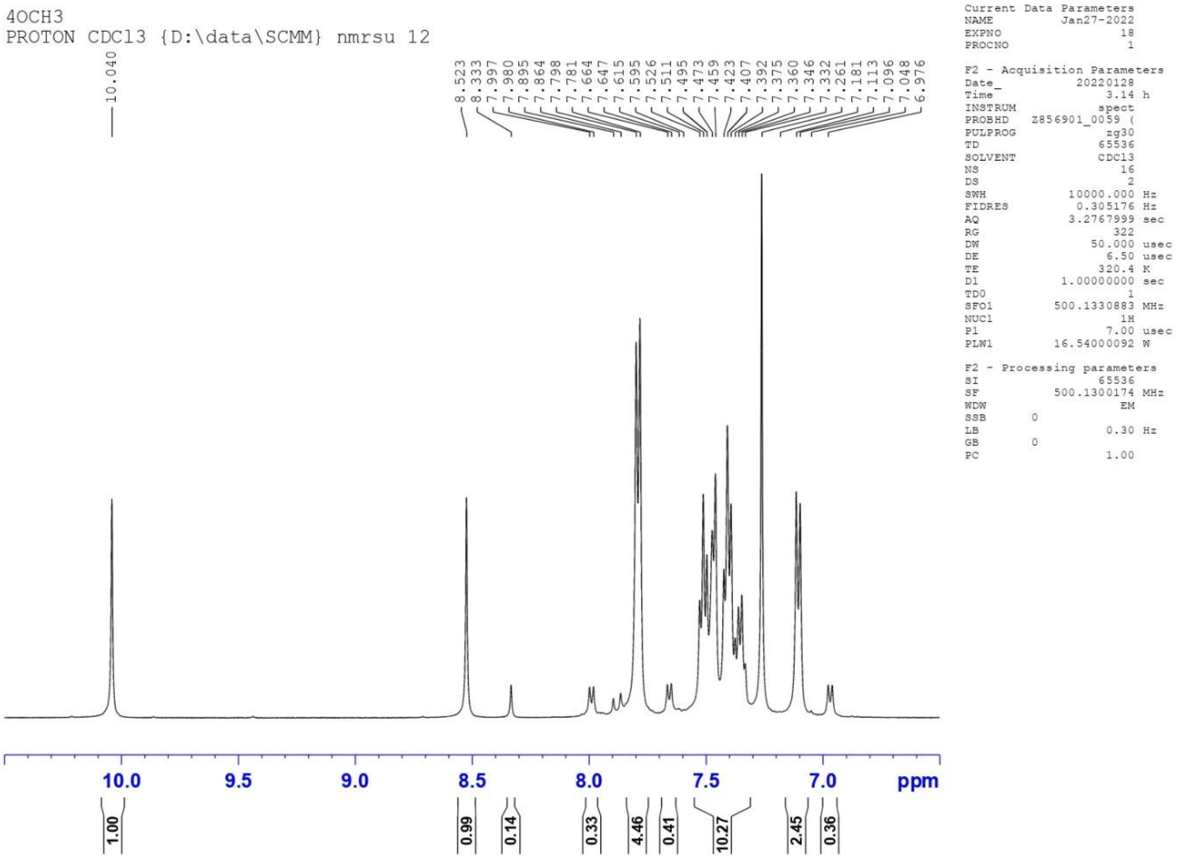

Figure S12. ^{1}H NMR of (E)-3-(3-(4-(benzyloxy)phenyl)-1-phenyl-1H-pyrazol-4-yl)-1-(4-methoxyphenyl)prop-2-en-1-one.

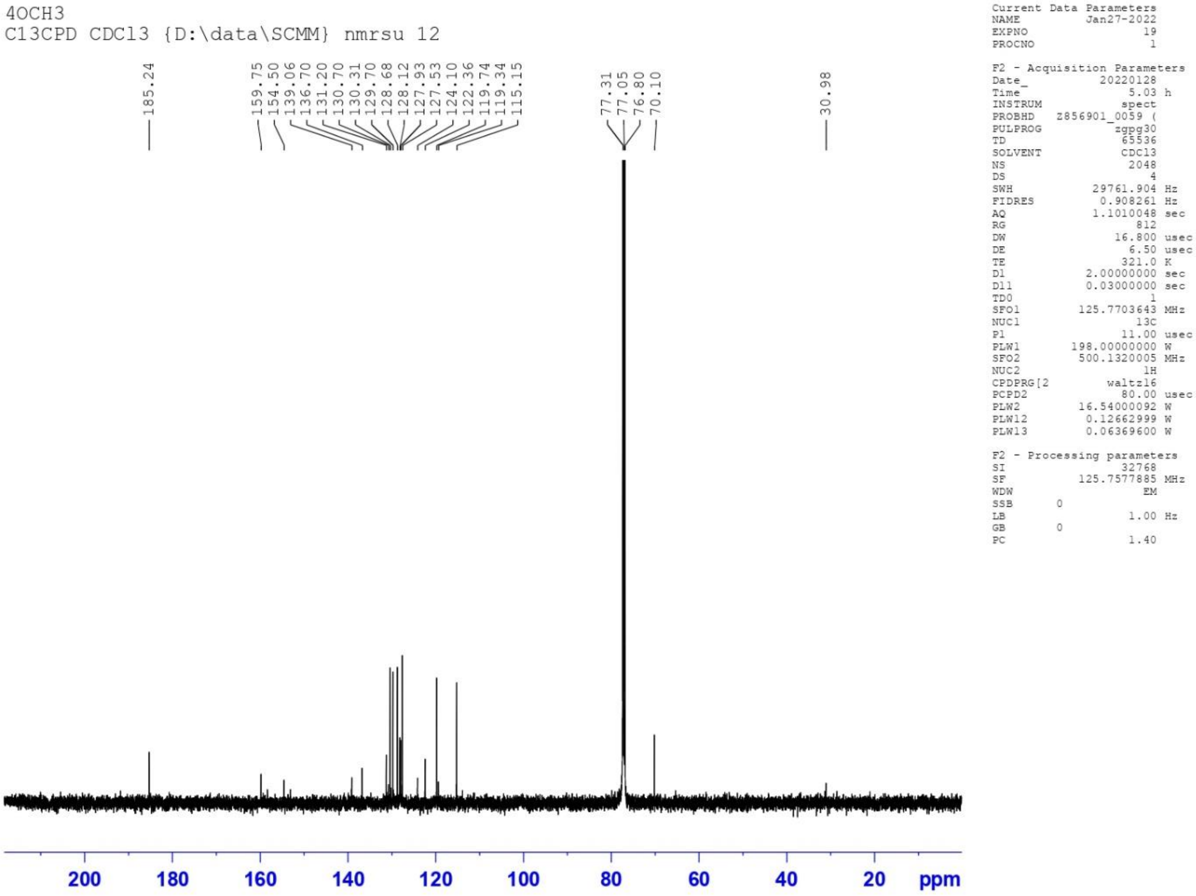

Figure S13. ^{13}C NMR of (E)-3-(3-(4-(benzyloxy)phenyl)-1-phenyl-1H-pyrazol-4-yl)-1-(4-methoxyphenyl)prop-2-en-1-one.

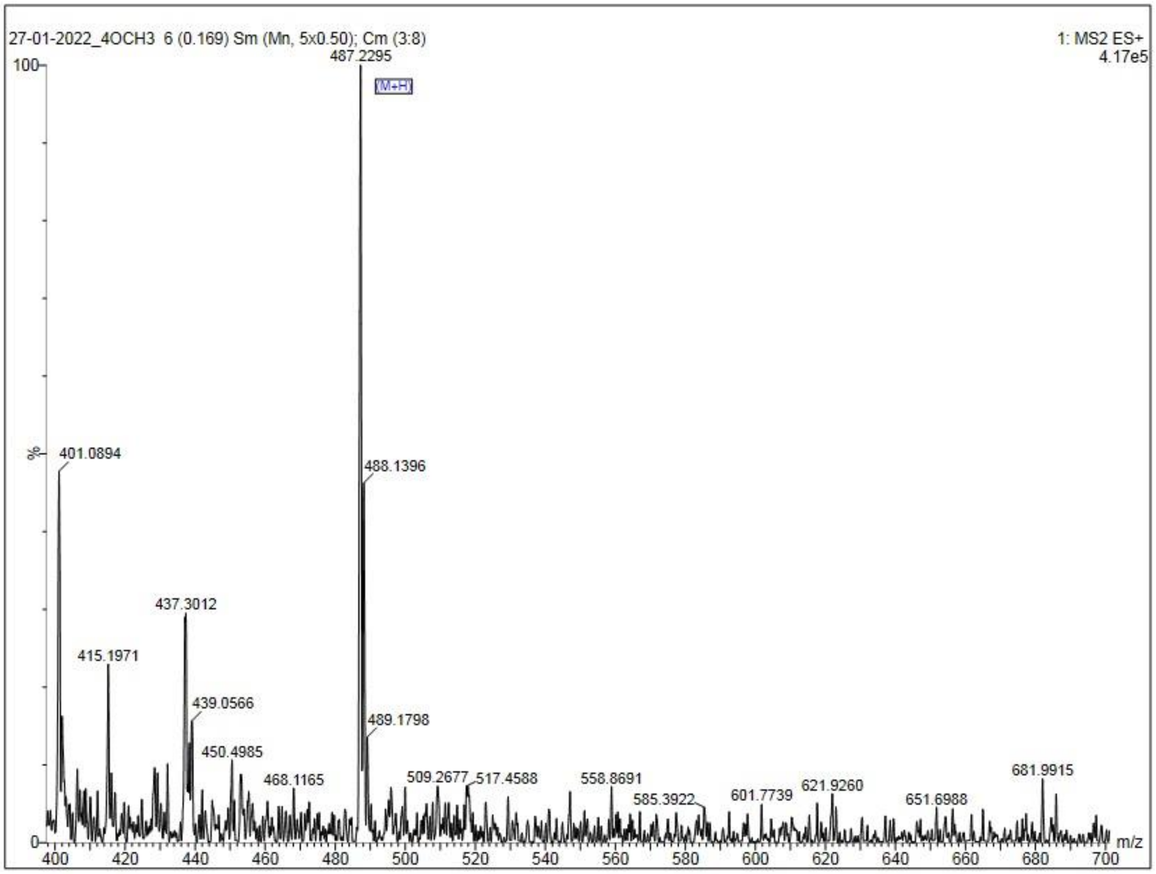

Figure S14. Mass spectra of (E)-3-(3-(4-(benzyloxy)phenyl)-1-phenyl-1H-pyrazol-4-yl)-1-(4-methoxyphenyl)prop-2-en-1-one.

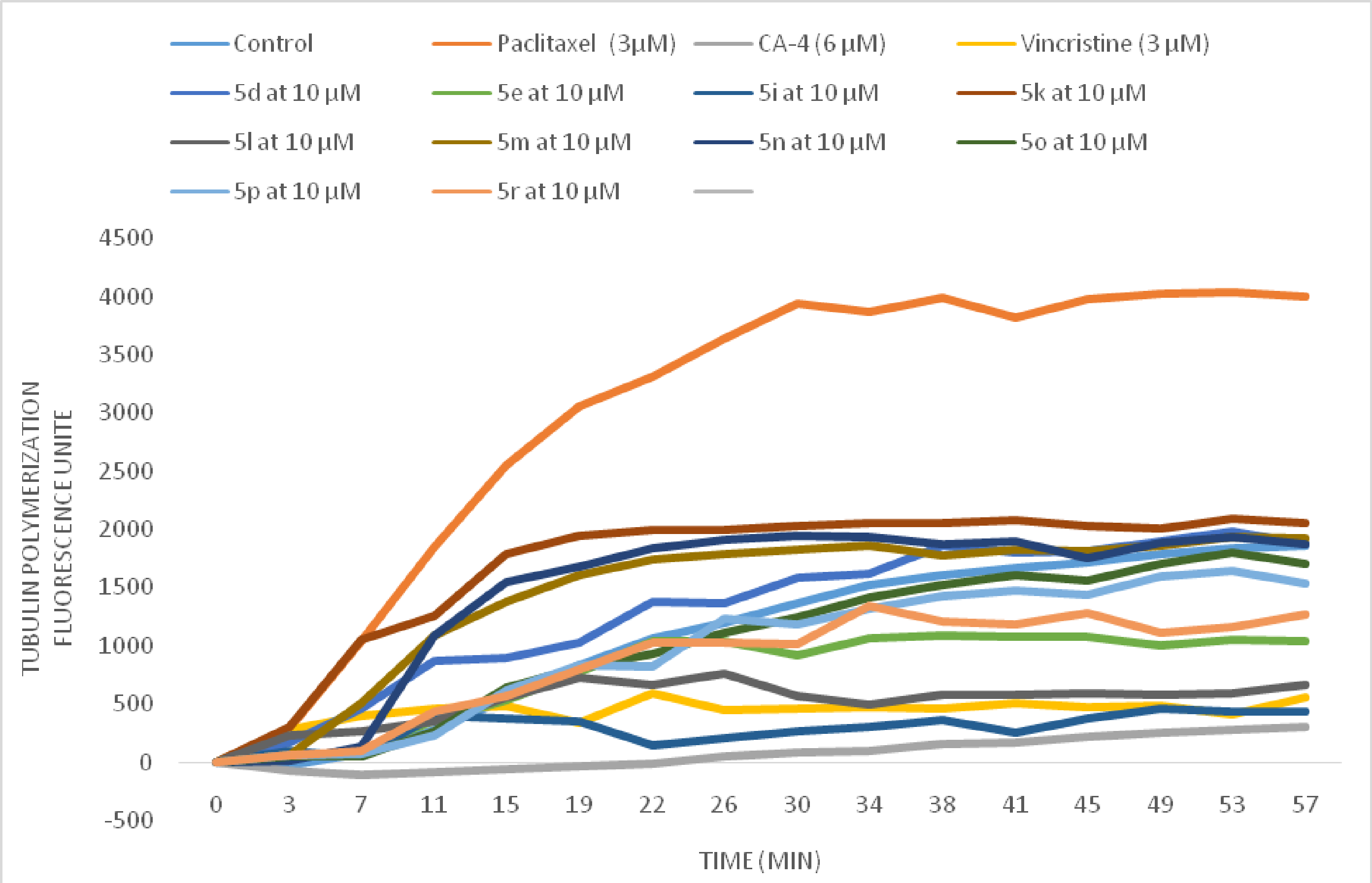

Figure S15. Effect of lead conjugates **5d, 5e, 5i, 5k, 5l, 5m, 5n, 5o, 5p** and **5r** on tubulin polymerization: Tubulin polymerization was monitored by the increase in fluorescence at 360 nm (excitation) and 420 nm (emission) for 1 h at 37 °C. Combretastatin A-4 was used as the reference standard in this study.
